# Barriers and facilitators to the implementation of guidelines in rare diseases: a systematic review

**DOI:** 10.1186/s13023-023-02667-9

**Published:** 2023-06-07

**Authors:** Matthew Gittus, Jiehan Chong, Anthea Sutton, Albert C. M. Ong, James Fotheringham

**Affiliations:** 1 Sheffield Kidney Institute, Sheffield Teaching Hospitals Trust, Sheffield, UK; 2 Academic Nephrology Unit, Department of Infection Immunity and Cardiovascular Disease, University of Sheffield Medical School, Sheffield, UK; 3 School of Health and Related Research, University of Sheffield, Sheffield, UK

## Abstract

**Background:**

Rare diseases present a challenge to guideline implementation due to a low prevalence in the general population and the unfamiliarity of healthcare professionals. Existing literature in more common diseases references barriers and facilitators to guideline implementation. This systematic review aims to identify these barriers and facilitators in rare diseases from existing literature.

**Methods:**

A multi-stage strategy included searching MEDLINE PubMed, EMBASE Ovid, Web of Science and Cochrane library from the earliest date available to April 2021, Orphanet journal hand-search, a pearl-growing strategy from a primary source and reference/citation search was performed. The Integrated Checklist of Determinants of Practice which comprises of twelve checklists and taxonomies, informed by 57 potential determinants was selected as a screening tool to identify determinants that warrant further in-depth investigation to inform design of future implementation strategies.

**Results:**

Forty-four studies were included, most of which were conducted in the United States (54.5%). There were 168 barriers across 36 determinants (37 studies) and 52 facilitators across 22 determinants (22 studies). Fifteen diseases were included across eight WHO ICD-11 disease categories. Together individual health professional factors and guideline factors formed the majority of the reported determinants (59.5% of barriers and 53.8% of facilitators). Overall, the three most reported individual barriers were the awareness/familiarity with the recommendation, domain knowledge and feasibility. The three most reported individual facilitators were awareness/familiarity with the recommendation, agreement with the recommendation and ability to readily access the guidelines. Resource barriers to implementation included technology costs, ancillary staff costs and more cost-effective alternatives. There was a paucity of studies reporting influential people, patient advocacy groups or opinion leaders, or organisational factors influencing implementation.

**Conclusions:**

Key barriers and facilitators to the implementation of clinical practice guidelines in the setting of rare diseases were at the individual health professional and guideline level. Influential people and organisational factors were relatively under-reported and warrant exploration, as does increasing the ability to access the guidelines as a potential intervention.

**Supplementary Information:**

The online version contains supplementary material available at 10.1186/s13023-023-02667-9.

## Background and objectives

Although rare diseases are individually rare, they are collectively common with an estimated global prevalence of 263–446 million people across 6000–7000 diseases [1, 2]. While a proportion of rare diseases have no accepted medical technologies, others have expensive therapeutic options with varying levels of evidence due to participant factors including small sample sizes leading to uncertainty, geographical dispersion and disease heterogeneity [3, 4]. Despite this, nearly six hundred orphan technologies to treat rare diseases have been approved by the Food and Drug Association in the US between 1983 and July 2020 with 552 on the market at the time of the NORD study [5]. A third of National Institute for Health and Clinical Excellence (NICE) approved technologies are for rare diseases [6]. These technologies have resulted in associated clinical practice guidelines, summarising up-to-date evidence and expert opinion leading to structured and practical recommendations to support decision making as prioritised by the World Health Organisation [4, 7].

The development and implementation of guidelines for rare diseases presents a greater challenge compared to more common diseases. This is related to limited health professional knowledge and experience in caring for those with specific rare diseases due to low disease prevalence [8]. These factors may lead to guideline adherence worse than the 30–70% non-adherence to guidelines reported in non-rare disease areas [9–12]. Frequently identified factors in existing systematic reviews for non-rare diseases include health professional level factors, a lack of knowledge [13], awareness of guidelines [13–15] and agreement with recommendations [13, 15]. Influencing factors at the organisational level include the absence of leadership/senior support [13, 16, 17], difficulties with teamwork [13, 17], disagreements with colleagues [13, 14] and insufficient communication [13].

Although there is a growing number of guidelines being published to inform the use of medical technologies for rare diseases, there is a paucity of systematic reviews or guidance on addressing the barriers and facilitators to the implementation of these recommendations in clinical practice [6, 18–20]. Such research is essential to ensure that people with rare diseases receive equitable high-quality healthcare. In this review, we aim to systematically identify and synthesise the factors influencing the implementation of clinical practice guidelines (CPGs) in the rare diseases setting. This will enable more informed development, implementation and evaluation of guidelines as well as the development of targeted interventions to improve implementation.

## Research design and methods

### Study design

We conducted a systematic review according to the Preferred Reporting Items for Systematic Review and Meta-analysis (PRISMA) statement. The study was registered on PROSPERO (CRD42021256061) then a protocol developed and published.

Studies were eligible for inclusion if they explored barriers and/or facilitators to the implementation of guidelines or consensus documents for rare diseases. Determinants of healthcare professional practice can be described as being factors that might prevent (barrier) or enable (facilitator) improvements in healthcare practice [21].

The European Union definition of a rare disease affecting less than 1 person per 2000 was used with prevalence confirmed on the Orphanet website [22]. Oncological rare diseases were excluded as they are predominantly managed by the oncology specialists rather than the related disease area. No restrictions were placed on the research design or publication date. As the study focuses on NICE technology appraisal guidance predominantly the results of the search strategy have been restricted to the English language. An overview of the inclusion and exclusion criteria is included in Table 1.

**Table 1:** Inclusion and exclusion criteria

Inclusion criteria	Exclusion criteria
Adults or paediatric patients	Non-English
Any stakeholder perspective	Non-rare disease
Established guideline or consensus document	No medication-based therapy
Rare disease as per Orphanet criteria	No established guideline or consensus document
Approved medication-based treatment or technology	

### Search strategy

A comprehensive search strategy, including database and supplementary techniques, was developed to maximise recall and reduce publication bias. An additional file includes the complete search strategy [see Additional file 1].

Search strategy stages:

- Stage 1: Rare diseases search
- Stage 2: NICE specialised technology appraisal search
- Stage 3: Orphanet Journal hand-search 28/02/17-28/02/21
- Stage 4: Pearl-growing subject search [23] from Denger et al. [24]
- Stage 5: Supplementary searches – grey literature, citations and references

#### Rare diseases search

The search strategy was developed using the SPIDER framework to identify studies with the expected study design, qualitative and mixed-methods (Table 2) [25]. Databases searched include MEDLINE via PubMed, EMBASE via Ovid, Web of Science and Cochrane Library from inception to April 2021.

**Table 2:** SPIDER tool

SPIDER elements	Keywords	Search terms and synonyms
S (Sample)	Health professionals	Health professional* or doctor* or clinician* or consultant* or GP or general practitioner* or physician* or pharmacist*
PI (Phenomenon of Interest)	Clinical practice guidelines for rare diseases	Guideline* or guidance or prescrib* or clinical protocol* or prescription* rare dis* or rare diagnos* or orphan dis*
D (Design)	Qualitative or mixed methodology	Ovid Medline qualitative search filter(23): (((“semi-structured” or semistructured or unstructured or informal or “in-depth” or indepth or “face-to-face” or structured or guide) adj3 (interview* or discussion* or questionnaire*)) or (focus group* or qualitative or ethnograph* or fieldwork or “field work” or “key informant”)).ti,ab. Or Interviews as topic/ or focus groups/ or narration/ or qualitative research/
E (Evaluation)	Influencing factors	Barrier* or facilit* or help* or hinder* or compliance or comply or complies or accept* or conform* or approv* or adhere* or strateg*
R (Research type)	Qualitative	Qualitative research/

#### NICE specialised technology appraisal search

NICE is an executive non-departmental public body of the Department of Health and Social Care in England and Wales that provides national guidance and advice to improve health and social care [26]. Published NICE guidelines and technology appraisals mandate the availability of technologies to people with rare diseases in England and Wales within three months, making their adoption in clinical practice less equivocal [27]. Furthermore, as the pharmaceutical industry often targets NICE first, then the rest of Europe, these approved medical technologies are likely to have generated the most peer reviewed literature.

**Fig. 1:**
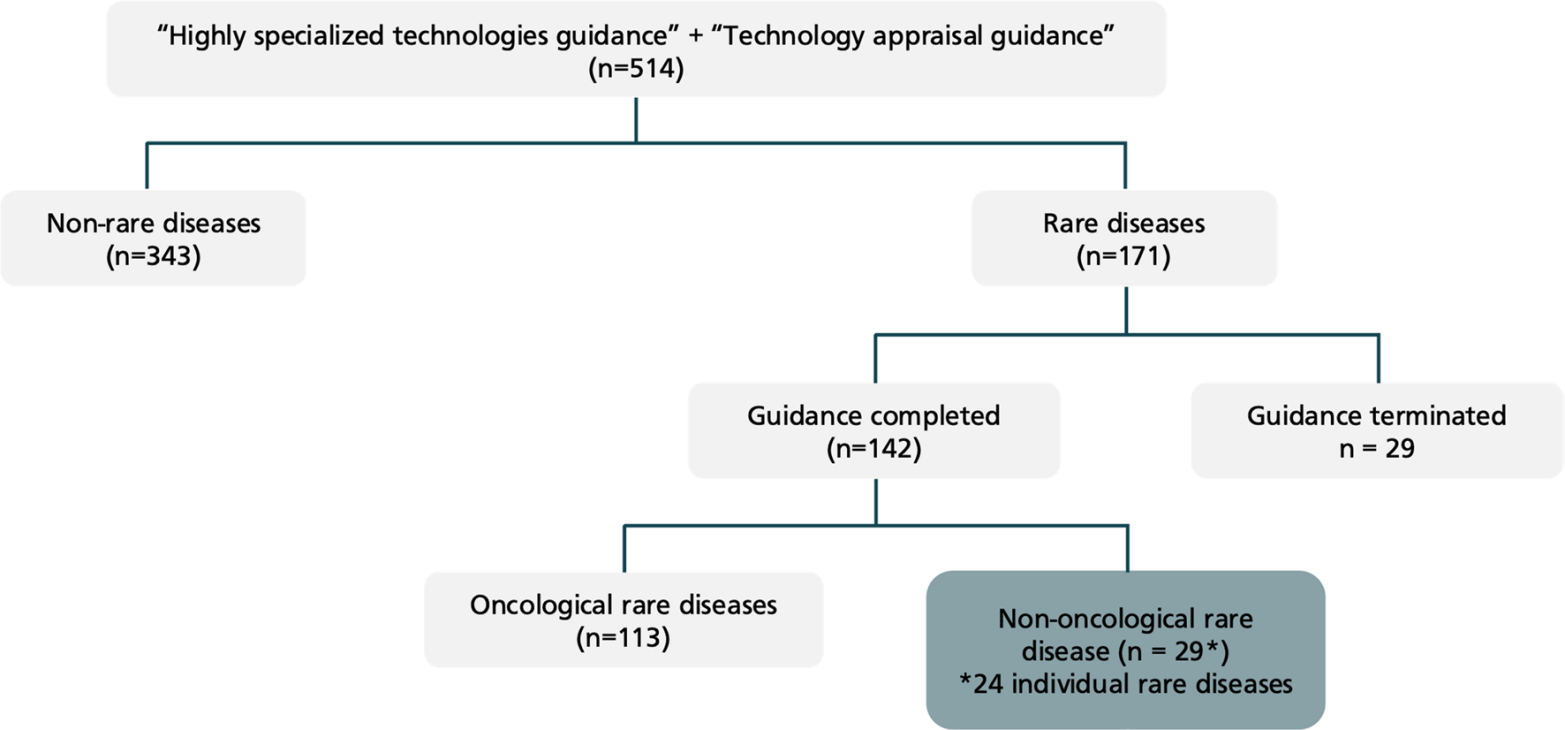
NICE technology appraisals to identify rare diseases with existing technology appraisal guidance

Published NICE technology appraisal guidance (TAG) and highly specialised technologies guidance documents were reviewed to identify non-oncological rare diseases with existing guidance on 27/02/2021 [6]. Twenty-nine current guidance documents were identified in twenty-four rare diseases as shown in Fig. 1 which were incorporated into the search strategy from the “Rare disease search”. An additional file includes the rare diseases identified [see Additional file 2]. As in the rare disease search, the database search included PubMed MEDLINE, Ovid EMBASE, Web of Science and Cochrane Library from inception to April 2021.

#### Orphanet Journal hand-search

A hand-search of the Orphanet Journal was implemented to locate studies that may not be found through traditional searches including those that may be unindexed in databases or informal publications [28, 29]. The Orphanet journal was selected for its high impact factor, relevance to the subject of the study, and publication of guidelines and conference proceedings on rare diseases. This involved a manual examination of the contents of the Orphanet journal editions between 28/02/17 to 28/02/21 by MG and JF. Five years was selected as the timescale as 75.9% of NICE technology appraisal guidance documents, we identified in the previously mentioned rare disease search, for non-oncological rare diseases were published between 2016 and 2020 [See Additional file 2]. Furthermore, the median time for the production of guidance for a NICE single technology appraisal is 48 weeks [30]. Thus, the results from journal issues for the preceding five years when the search was undertaken in February 2021 should account for the time for NICE technology appraisal guidance publication, development and research into implementation in clinical practice.

#### Pearl-growing subject search

Through the “Rare disease search” we identified a primary manuscript published by Denger et al. (2019) that explored the barriers and facilitators to guideline adherence for a specific rare disease, Duchenne’s Muscular Dystrophy [24]. We developed a subject pearl-growing strategy using the Medical Subject Headings (MeSH) terms indexed for the study published by Denger et al. (2019). The MeSH term (guideline adherence*) was combined with the non-oncological rare diseases identified to have current NICE TA guidelines to search PubMed MEDLINE i.e. (*rare disease*) AND (guideline adherence*). The decision for the pragmatic search using PubMed Medline was suitable due to the comprehensiveness of the overall search strategy and the specificity of MeSH terms.

#### Supplementary searches

Grey literature was obtained through discussion with the NICE Health Technology Adoption team who support the uptake of new technologies recommended by NICE through system learning based on usage and clinical engagement data. Data sources were sought from this group given their experience in engaging with our stakeholders as well as the identification of obstacles and solutions to technology adoption in clinical practice. An additional file includes the grey literature provided [see Additional file 1].

References and citations of all included studies were hand-searched and assessed for suitability for inclusion with repeated cycles until no further studies were identified.

### Study selection and data extraction

Following the elimination of duplicates, two reviewers (MG and JF) reviewed the titles and abstracts according to the inclusion criteria. The full-text review was conducted by two independent reviewers (MG and JC) with any disagreements resolved through discussion and a consensus reached. Reasons for exclusion were recorded on the data extraction template.

Data extraction was developed by reviewers then piloted and undertaken. Information included authors, publication year, database ID, location, ICD-11 disease category, study design, type of participant and number of responses.

### Quality assessment

To ensure transparency, all included studies were appraised using best practice quality appraisal tools relevant to their specific research design, Table 3. All appraisals were conducted by MG and verified by JF.

**Table 3:** Quality assessment tools

Quality assessment tool	Abbreviation	Type of study
Joanna Briggs Institute – Text & Opinion	JBI-TO	Review article
Quality improvement – Minimum Quality Criteria Set	QI-MQCS	Quality improvement project
Critical Appraisal Skills Programme – Qualitative studies	CASP	Qualitative study
Mixed Methods Appraisal Tool	MMAT	Mixed methods study
Risk Of Bias Instrument for Cross-Sectional Surveys of Attitudes And Practices	ROBICSSAP	Survey/questionnaire
Risk Of Bias In Systematic Reviews	ROBIS	Systematic reviews

### Data analysis and synthesis

Thematic analysis was performed using the Integrated Checklist of Determinants of Practice as this framework was specifically developed for healthcare improvement [21]. The checklist was formed through the aggregation of the components from twelve existing checklists, frameworks and taxonomies for chronic diseases which were identified through a systematic review process. It consists of fifty-seven determinants grouped into seven domains (guideline factors, individual health professional factors, patient factors, professional interactions, incentives and resources, capacity for organisational change, and social, political and legal factors). The determinants can be interpretated as barriers or facilitators and are sufficiently diverse and detailed to encompass factors identified in the included studies. Due to the heterogeneity of questions and study design, statistical aggregation was not appropriate.

## Results

**Fig. 2:**
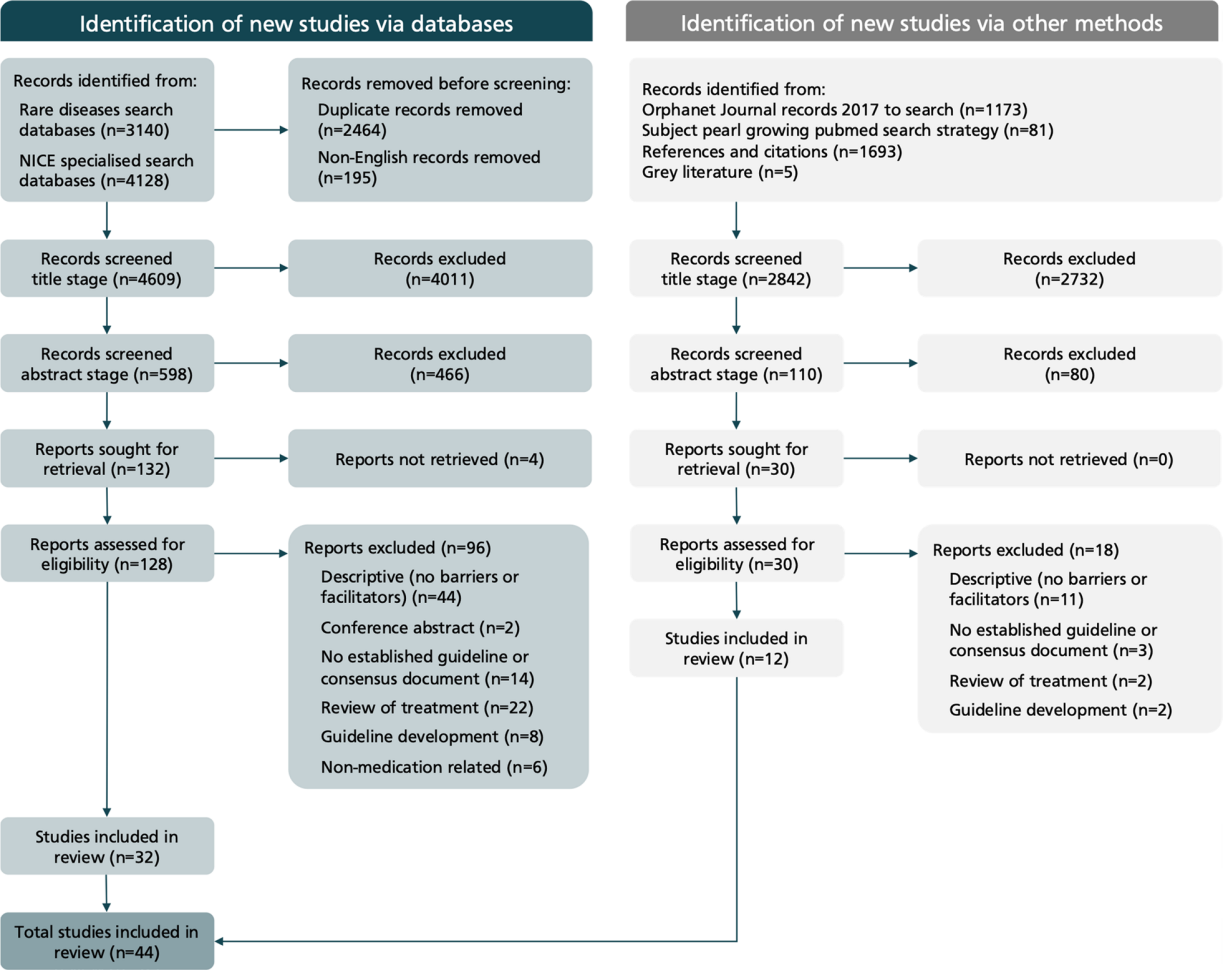
PRISMA flow chart

After eliminating duplicates 7548 titles were identified. 158 studies were selected for full-text review and 44 were included in the thematic synthesis (using the determinants of practice in the ICDP framework). The PRISMA flow chart summarising the review process is in Fig. 2. [31] Additional files show the full multi-stage PRISMA flow chart [see Additional file 3] and the studies excluded at the full-text stage [see Additional file 4].

Most included studies were conducted in the United States (54.5%) with the remaining studies being multi-national or from countries with a high Human Development Index [32]. Publication dates ranged from 1995 to 2021 (median 2016) with an increasing trend in publication rate when studies from 2020 and 2021 were excluded (overall reduction in research due to COVID-19 pandemic). There were fifteen rare diseases across eight WHO ICD-11 categories [33] in the included studies with diseases of the immune system accounting for a quarter (Table 4). An overview of the included studies is available as an additional file [see Additional file 5].

**Table 4:** WHO ICD-11 disease categories and rare diseases of the included studies

ICD-11 disease categories	Orphanet prevalence	No. of studies
**03: Diseases of the blood or blood-forming organs**		**10 (22.7%)**
Atypical Haemolytic Uraemic Syndrome	1–9/100,000	1
Sickle Cell Disease	1–5/10,000	9
**04: Diseases of the immune system**		**11 (25.0%)**
Hereditary Angioedema	1–9/100,000	6
Primary Immunodeficiency	1–9/100,000	5
**05: Endocrine, nutritional or metabolic disease**		**2 (4.5%)**
Urea Cycle Disorders (group of disorders)	–	1
Gaucher’s disease	1–9/100,000	1
**08: Diseases of the nervous system**		**6 (13.6%)**
Duchenne Muscular Dystrophy	1–9/100,000	5
Spinal Muscular Atrophy (group)	–	1
**09: Diseases of the visual system**		**2 (4.5%)**
Rare Non-Infectious Uveitis (group)	–	2
**12: Diseases of the respiratory system**		**10 (22.7%)**
Cystic Fibrosis	1–5/10,000	4
Idiopathic Bronchiectasis	–	1
Idiopathic Pulmonary Fibrosis	1–5/10,000	5
**13: Diseases of the digestive system**		**1 (2.3%)**
Primary Biliary Cholangitis	1–5/10,000	1
**15: Diseases of the musculoskeletal system or connective tissue**		**1 (2.3%)**
Rare Connective Tissues Disease (group)	–	1
**Not applicable**		**1 (2.3%)**
Non-specific	NA	1

ICD-11 Mortality and Morbidity Statistics codes are indicated in bold

*Disease prevalence when Orphanet database searched on 22/02/2022 [109]

Five studies (11.4%) included interviews or focus groups in their design compared to thirty-three studies (75.0%) that incorporated questionnaires or surveys. Most studies reported the perceptions or experiences of respondents (n = 35, 79.5%) rather than retrospective chart review (n = 6, 13.6%) or expert opinion (n = 3, 6.8%). Non-highly specialised health professionals were the most common respondent type (n = 21) compared to highly specialised health professionals (n = 14) and non-health professionals (n = 5). An additional file has the full description of the included studies [see Additional file 5]. Studies rated as having a higher risk of bias in their specific quality appraisal tool were only included where their identified determinants of practice were supported by other studies included in the review with a low risk of bias. An additional file includes further details of the quality assessment [see Additional file 6].

### Determinants of practice – barriers and facilitators

In accordance with the definitions used by the ICDP, determinants are considered barriers if their presence impedes the implementation of or adherence to rare disease guideline(s). In contrast, they are considered facilitators if their presence promotes the implementation of or adherence to the rare disease guideline(s) [16]. We considered a determinant as neutral when it could be interpreted as having a positive or negative impact.

**Fig. 3:**
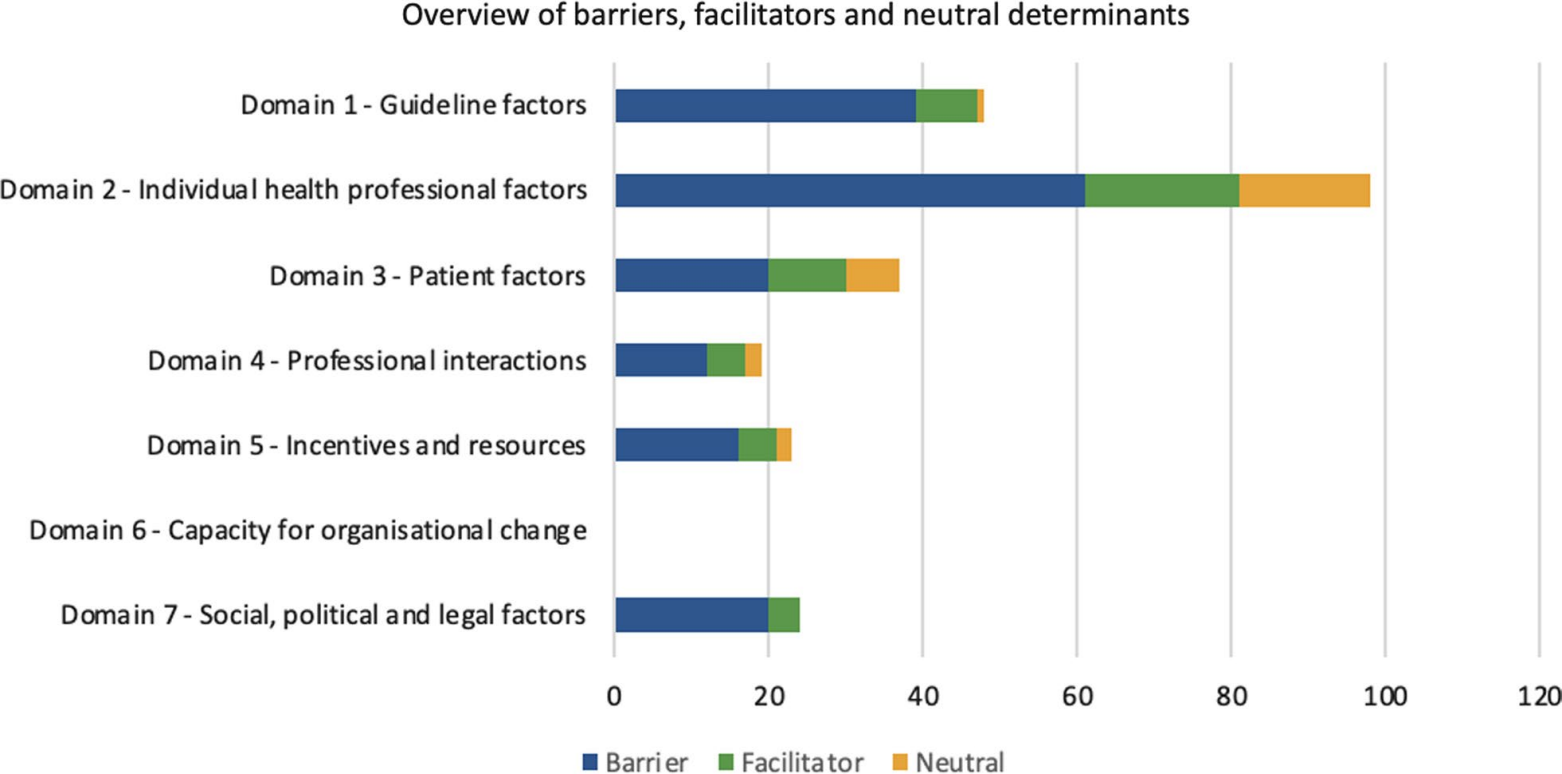
Stacked bar chart of barriers, facilitators and neutral determinants across the seven determinants of the ICDP

The data synthesis produced 168 examples of reported barriers from 37 studies corresponding to 36 determinants in the ICDP and 52 examples of reported facilitators from 22 studies corresponding to 22 determinants in the ICDP. Figure 3 and Table 5 summarise identified factors with a comprehensive analysis in additional files [see Additional files 7 and 8].

**Table 5:**
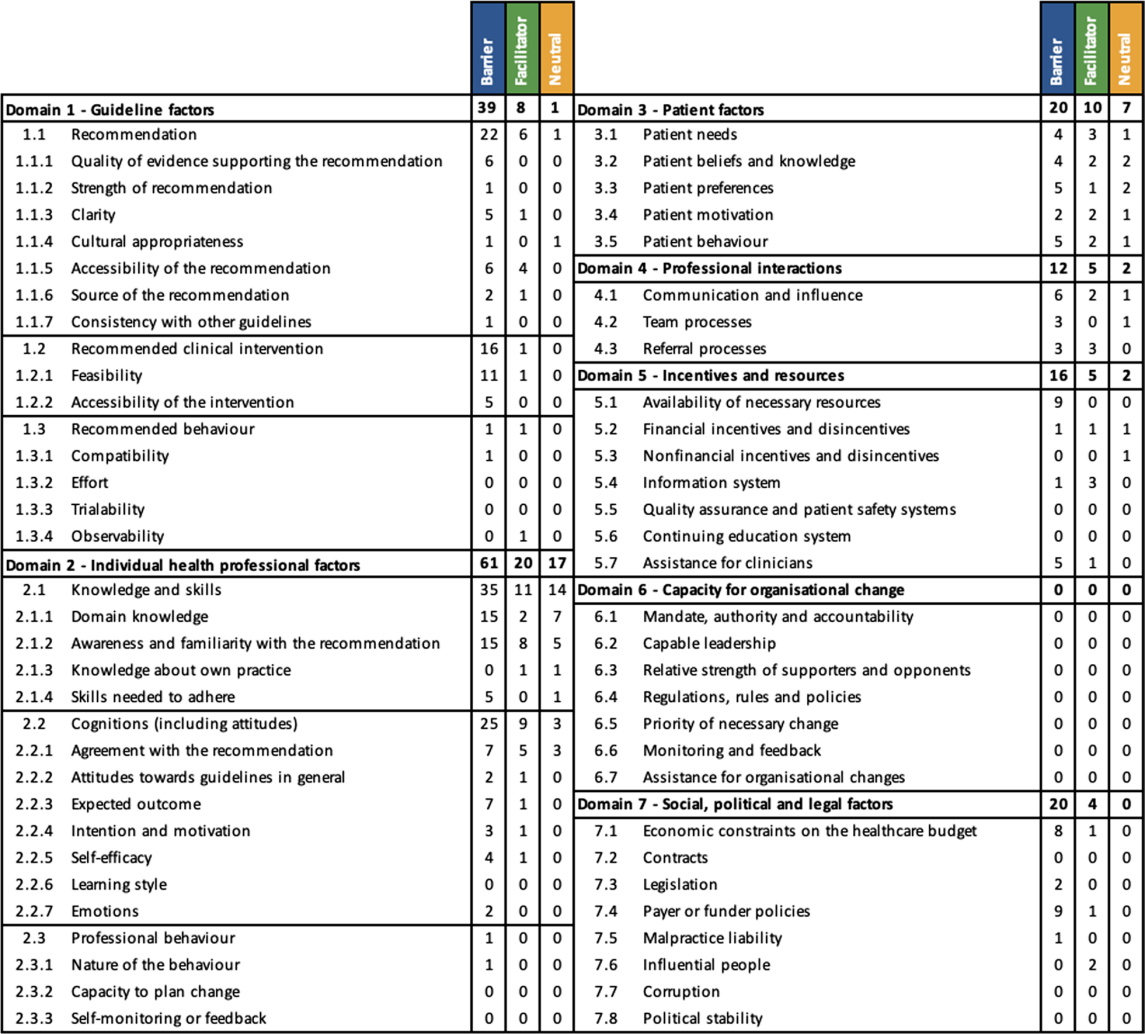
Abbreviated overview of barriers, facilitators and neutral determinants influencing guideline implementation across the seven domains of the ICDP

The individual health professional factors domain was the most prevalent domain. Awareness and familiarity with the recommendation (determinant 2.1.2) was the most reported individual determinant of practice (Table 6). An additional file includes the contribution of individual studies to the determinants of practice [see Additional file 9].

**Table 6:** Top 3 determinants identified in included studies

Top 3 barriers	Top 3 facilitators
Awareness and familiarity with the recommendation (n = 15)	Awareness and familiarity with the recommendation (n = 8)
Domain knowledge (n = 15)	Agreement with the recommendation (n = 5)
Feasibility (n = 11)	Accessibility of the recommendation (n = 4)

#### Guideline factors

The quality of evidence, clarity and feasibility of the recommendation were the highest reported determinants in this domain potentially limiting the implementation of guidelines in the included studies. This included a lack of sufficient evidence [24, 34–38] and dependence on expert opinion [39]. Clarity of guideline recommendations was considered to facilitate implementation through avoidance of jargon, lengthy and text-heavy guidance [35, 40], and clear indications for initiation [36, 38]. The included studies reported difficulties retrieving guidelines [41–43], poor dissemination [44] and insufficient translation to other languages [41].

Feasibility of recommendations influences the likelihood of implementation through as less feasible recommendations are perceived to require more time to implement [44–47] and less convenient for both patients and healthcare professionals [48, 49]. This may be related to perceived suitability of recommendations for healthcare in practice [24, 37, 40, 44, 50] and adaptability of the recommendations to different healthcare systems [36, 44]. The accessibility of recommended interventions also presents an obstacle to implementation requiring sufficient technology access/fluency [40, 50], access to investigations [42, 51] and alternatives being more accessible/feasible [46, 52].

Supporting information technology was cited as a facilitator to implementation of guidance by three studies through the use of mobile apps [53], guidelines applications [40] and electronic medical records [54]. Insufficient digital resources impair guideline dissemination leading to under-utilisation [55]. Systems tracking prescribing adherence may also improve adherence to recommended interventions and the quality of care delivered to patients [56].

#### Individual health professional factors

Awareness and familiarity with the recommendations were reported to influence implementation in a large number of included studies [34, 36, 38–40, 42–45, 50, 51, 53–55, 57–66]. This was present in studies involving highly specialised health professionals (n = 6, 13.6%) as well as non-highly specialised health professionals (n = 22, 50%). All non-highly specialised health professionals were trained in the same disease area or could be expected to implement the recommendations for rare disease patients. Low frequency of encountering patients with the specific rare disease was reported as a potential reason for limited awareness/familiarity [24, 39]. Some studies included suggestions to improve awareness including education [43, 59], inaccessibility [53], regional network and awareness campaigns [42].

Health professionals’ knowledge in the rare disease subject area (domain knowledge) limited the implementation of recommendations in many studies [24, 36, 43, 44, 47, 48, 50, 54, 56–58, 61, 67–70]. Domain knowledge is recognised as important in the management of patients, awareness of the guidance and recognition of their importance [24, 54]. Other aspects of knowledge and skills required for guideline implementation included the lack of specific training [24, 37, 47, 67], dedicated education sessions or materials [50, 56, 69] and associated skills [36, 38]. Unsurprisingly there was a reported difference between different types of healthcare professionals with highly specialised health professionals having greater knowledge of treatment options and guidelines when compared to broader clinical experience such as primary care physicians [46, 61, 66, 70]. Furthermore, general experience as a healthcare professional and specifically experience managing patients with the rare disease in question were considered as a positive factor leading to increased adherence to guidelines [24, 48, 49, 58, 67].

The perception of guidelines by health professionals may explain some variation in practice through the agreement with using guidelines in clinical practice [36, 43] or agreement with the specific recommendations [36, 38, 39, 43, 44, 46, 51, 55, 60, 61, 71, 72]. Outcome expectancy impaired the implementation when it was perceived that the recommendation would not affect patient outcomes [38, 39, 55] or that health professionals anticipated poor patient compliance [55, 61], expected adverse outcomes [67] or that recommendations may cause anxiety to patients [46]. Attitudes and emotions of health professionals were found to negatively affect adherence to recommendations in Sickle Cell Disease (SCD) care related to perceived opiate-seeking behaviour [34, 67, 73].

Health professional self-reported capability (efficacy) in managing patients with a rare disease limited implementation, non-rare disease specialists feel unable to provide care [53], adhere to recommendations [43] or interpret outcomes of recommendations [55]. This is potentially coupled with professionals’ failure or delays in prescribing recommended therapies [61].

#### Patient factors

Patient needs or demands of their healthcare providers were reported to potentially influence guideline implementation. These factors included the home-to-clinic distance for patients [45], perceived additional costs to patients [39, 67] and unrealistic patient expectations [37]. Some studies suggested that implementation of recommendations could be supported through recognising patient needs and developing guidelines in a patient-centred method [24, 44, 54].

Patient knowledge and beliefs were recognised as a barrier through patients being unaware of the need to attend for recommended interventions [74]. Patient and caregiver unawareness of the guidelines or disease knowledge was identified as a factor that may limit their engagement and potentially impede guideline implementation [24, 40, 44, 45, 47].

Patient preferences for the location of their care [24], patient-focussed priorities [24, 37, 46] and avoidance of additional treatment burden [36] were reported to limit the implementation of some recommendations. This could manifest in an “adversarial” manner through poor medication adherence [36, 67, 73] or low outpatient attendance [39, 67]. This could be in part related to unvoiced disagreements with the healthcare professionals responsible for their care [40]. However, engaged patients or relatives can make implementation of recommendations easier and reduce social stigma [24, 56]. In fact, McPhail et al. (2010) recommend sharing the guidelines with patients and their families to empower them and improve adherence to guidance in the chronic care setting [56]. Denger et al. (2019) suggest that patients adhering to recommendations may encourage other patients with the same disease to adhere to recommendations as a form of peer pressure. They also propose that recommendations that do not interfere with patients’ everyday life are more likely to have better adherence [24]. Interpersonal relationships between health professionals and patients/caregivers have been suggested to influence patients’ motivation and willingness to participate in care [75]. A patient’s motivation could then impede implementation for example people with SCD have described being demotivated to attend hospital for fear of being perceived to be drug-seeking and facing potential discrimination [67, 73].

#### Professional interactions

Some studies described poor communication and coordination between primary and secondary care potentially impeding the implementation of recommendations [45, 47, 53, 54, 75]. These findings contrast those of Heutinck et al. (2021) who reported that surveyed physicians were satisfied with the inter-professional communication about Duchenne Muscular Dystrophy patient care although reasons for this outlying study were not explored further [39]. Referral processes were believed to be underlie some of these inter-professional communication difficulties including practical difficulties [54], lack of awareness of processes [57] or insufficient information on referrals [52]. Other authors have supported this by reporting that good referral pathways improve the care of patients with rare diseases, guideline adherence and the education of non-specialists healthcare professionals [50, 73, 75].

#### Financial incentives and resources

Availability of resources and financial disincentives were found to impair guideline implementation. Specific reasons for the reduced availability of necessary resources and financial considerations included unavailable/insufficient therapies [39, 42, 49, 75], health technology costs for the recommended intervention [24, 46, 75], inappropriate clinical spaces/schedules [45, 55], ancillary staff costs [39, 45], general costs [24, 36] and inadequate time [39, 45, 54]. More cost-effective alternative preparations may also impede adherence to recommendations in guidelines [47]. Utrankar et al. (2018) suggest financial incentives or penalties can improve the completion of guideline-derived objectives [40]. Insufficient support staff was believed to impair the ability of clinicians to comply with recommendations through poor coordination [39] and resource management [43].

#### Capacity for organisational change

The capacity for organisational change was not a feature of the included studies. This could be related to the predominantly patient or health professional focus of these studies which would not involve in-depth assessment of organisational factors.

#### Social, political and legal factors

The main hurdles described by the studies at the social, political and legal level were costs and payer or funder policies. Economic constraints can influence the funds available for recommended treatments [75, 76], dosage prescribed [76], ancillary staff [45], capacity of services [39] and overall ability to adhere to recommendations [24, 36, 48, 49, 75]. Proposed mechanisms for payer or funder policies influencing guideline implementation included insufficient insurance coverage [48, 49, 67, 69] and difficulties obtaining reimbursement [36, 44, 56, 69]. Masese et al. (2019) described respondents believing that their ability to deliver good care was not influenced by insufficient insurance coverage. However, they did not specifically enquire about whether it influenced their ability to follow recommendations or patients’ behaviours [54].

There were limited descriptions of influential people in the studies. Banerji et al. (2016) acknowledge the role of patient advocacy groups and patient representatives in improving the uptake of recommendations from guidelines for patients with hereditary angioedema [50]. Behr (2016) recognises “powerful personalities or groups” as potentially supporting evidenced-based guidance as well as mis-information or over-information illustrating that the involvement of influential people is not always positive [35].

Both patient groups and opinion leaders and patient groups have been recognised as having a role in guideline implementation for more common diseases [77, 78].

## Discussion

This systematic review identified, quality appraised and synthesised forty-four studies assessing factors influencing clinical practice guideline implementation. There has been increased publication of studies assessing guideline implementation over the last twenty years prior to the COVID-19 pandemic. Like others, we found that the most frequently cited barriers were at the level of the individual health professional [13–15, 77], with the awareness and familiarity of health professionals being the most common barrier. Although rare diseases are often considered the sole domain of healthcare professionals with highly-specialised expertise [79], our review identified that the majority of research had been performed in non-specialists. There are often only a handful of specialists in a country, or even worldwide, who have expertise in a given rare condition [80–82]. A National Organization for Rare Diseases survey in 2020 showed that 20% of respondents were not being managed by a specialist [82], which has previously been associated with inappropriate treatment and worse patient outcomes [83–85]. This is may be explained through recognised difficulties in non-highly specialised health professionals gaining adequate experience due to low patient prevalence [86]. These factors are also frequently recognised by many rare disease organisation strategies including the UK Rare Diseases Framework [87], EURODIS: Recommendations from the Rare 2030 Foresight Study [88], Canada’s Rare Disease Strategy [89] and the Australian National Strategic Action Plan for Rare Diseases [90].

Our study identified feasibility of guideline implementation as a barrier, and it has been noted that national guidelines often lack details of the applicability and description of the changes needed to apply recommendations [91–93]. The customisation of clinical practice guidelines to particular organisations or healthcare systems is already in practice for the management of cancer in France [94], and may lead to better adherence and outcomes. Our study also found health professionals’ anticipated poor adherence to therapies by the patient, which has been shown to be lowest in patients who were asymptomatic and younger [95], is associated with worse outcomes and increased healthcare costs [96], and can be improved by enhancing social support from healthcare professional and providers [97, 98]. Medication adherence is important due to the risk of worsening disease, death and increased health care costs.

Although, key opinion leaders and influential people have a role in the development of new technologies, and the development and adherence to new policies and guidelines [99], we found a paucity of them in our included studies. An opinion leader who is an individual that is perceived as credible, trustworthy and able to exert influence on others’ decision-making [100]. In the wider healthcare setting, opinion leaders are been proposed to improve health professionals’ familiarity, knowledge and compliance to recommendations and knowledge [77, 101]. Furthermore, opinion leaders have been considered as an effective strategy for the implementation of research findings in specialised areas such as rare diseases [102].

### Strengths and limitations

The strengths of this review include the aggregation of determinants of practice from different regions, healthcare settings and rare diseases supporting the generalisability of findings. However, it is important to recognise the limitations of this approach as it may be difficult to incorporate all findings into the classification of the framework. For example, it is not possible to incorporate any factors that may encompass social influence principles such as social proof or commitment [103].

Barriers and facilitators examined were to recommendations from the NICE organisation, based in England & Wales, and other international organisations publishing guidelines for use the care of people with rare diseases. Synthesis was supported by a range of existing systematically developed, validated and peer-reviewed tools.

### Recommendations for clinical practice and future research

Future guidelines should involve key opinion leaders, patient advocacy groups and people with the rare disease, and consider modifying any relevant specific determinants of practice that recommendations could be affected by. This could be achieved through modelling a single disease to produce contextually appropriate targets and sustained change.

Deeper understanding of the factors influencing guideline implementation for rare diseases could be achieved by future studies focussing on underlying theoretical principles such as social proof, commitment, self-efficacy, outcome expectations and other beliefs, and may be achieved using ethnographic research [104–106]. Only used by five studies in our review, there are limitations to ethnographic approaches in the study of rare diseases due to a limited number of potential participants (patients and healthcare professionals), perceived high cost and a potential lack of generalisable findings [107]. However, the concept of generalisability itself has been argued by some researchers to not be the purpose of qualitative research with a greater focus on depth of understanding within a study’s specific context [108].

## Conclusions

In this review we identified forty-four studies focusing on the barriers and facilitators to guideline implementation in the rare disease setting. All the studies included were from countries with a higher human development index. It combines findings from both highly specialised and non-highly specialised health professionals. The synthesis included 168 reported barriers and 52 reported facilitators with the individual health professionals domain being the most common. Influential people as a facilitator for guideline implementation was surprisingly absent given the role of this stakeholder in other aspects of rare disease guideline development and research. Capacity for organisational change was relatively under-reported which may be related to the limited number of ethnographic studies in the literature available. Future research and guideline implementation strategies should focus on the most commonly reported determinants in this study.

## Supplementary Information

**Additional file 1.** Search strategy.

**Additional file 2.** Approved NICE technology appraisal guidance for non-oncology rare diseases.

**Additional file 3.** PRISMA flow diagram.

**Additional file 4.** List of excluded studies at full-text stage.

**Additional file 5.** Included study characteristics.

**Additional file 6.** Risk of bias assessments.

**Additional file. 7** Determinants of Practice.

**Additional file. 8** Granular infographic of determinants of practice distribution.

**Additional file 9.** Overview of the contribution of individual studies.

## Data Availability

All data generated or analysed during this stage are included in this published article [and its Additional files].

## Abbreviations

CPG: Clinical Practice Guideline
ICDP: Integrated Checklist of Determinants of Practice
NICE: National Institute for Health and Care Excellence
NORD: National Organization for Rare Diseases
PAGs: Patient Advocacy Groups
RDCRN: Rare Disease Care Research Network
SCD: Sickle Cell Disease
TA: Technology Appraisal
TA: Technology Appraisal Guidance
WHO: World Health Organisation
