# Supplementary material for: Barriers and facilitators to the implementation of guidelines in rare diseases: a systematic review
Source: Orphanet J Rare Dis. 2023 Jun 7;18:140. doi: 10.1186/s13023-023-02667-9 (PMC10246545; doi:10.1186/s13023-023-02667-9)
Supplement: Supplementary file 1 — Additional file 1. Search strategy. [file 13023_2023_2667_MOESM1_ESM.docx]

## **Additional file 1 – Search strategy**

## **Rare disease search**

### **MEDLINE**

Database: Ovid MEDLINE(R) and Epub Ahead of Print, In-Process, In-Data-Review & Other Non-Indexed Citations and Daily <1946 to March 25, 2021>

Date Run: 02/04/2021 Results: **839**

| #1 | exp Rare diseases/ |
| --- | --- |
| #2 | (rare dis* or rare diagnos* or orphan dis*).mp. |
| #3 | 1 or 2 |
| #4 | exp Technology Assessment, Biomedical/ |
| #5 | (NICE or “national institute for health and care excellence”).mp. |
| #6 | Drug Prescriptions/ |
| #7 | (guideline* or guidance or prescrib* or clinical protocol* or prescription*).mp. |
| #8 | 4 or 5 or 6 or 7 |
| #9 | 3 and 8 |
| #10 | (barrier* or facilit* or help* or hinder* or compliance or comply or complies or accept* or conform* or approv* or adhere* or strateg*).mp. |
| #11 | (((“semi-structured” or semistructured or unstructured or informal or “in-depth” or indepth or “face-to-face” or structured or guide) adj3 (interview* or discussion* or questionnaire*)) or (focus group* or qualitative or ethnograph* or fieldwork or “field work” or “key informant”)).ti,ab. or Interviews as topic/ or Focus groups/ or Narration/ or Qualitative research/ |
| #12 | (health professional* or doctor* or clinician* or consultant* or GP or general practitioner* or physician* or pharmacist*).mp. |
| #13 | 10 or 11 or 12 |
| #14 | 9 and 13 |

### **COCHRANE**

Date Run: 13/04/21 Results: **739**

| #1 | MeSH descriptor: explode all trees |
| --- | --- |
| #2 | (rare dis* or rare diagnos* or orphan dis*):ti,ab,kw |
| #3 | #1 or #2 |
| #4 | MeSH descriptor: [Technology Assessment, Biomedical] explode all trees |
| #5 | (NICE or "national institute for health and care excellence"):ti,ab,kw |
| #6 | MeSH descriptor: [Drug Prescriptions] explode all trees |
| #7 | (guideline* or guidance or prescrib* or clinical protocol* or prescription*):ti,ab,kw |
| #8 | #4 or #5 or #6 or #7 |
| #9 | #3 and #8 |
| #10 | (barrier* or facilit* or help* or hinder* or compliance or comply or complies or accept* or conform* or approv* or adhere* or strateg*):kw,ab,ti |
| #11 | ((("semi-structured" or semistructured or unstructured or informal or "in-depth" or indepth or "face-to-face" or structured or guide) NEAR/3 (interview* or discussion* or questionnaire*)) or (focus group* or qualitative or ethnograph* or fieldwork or "field work" or "key informant")):ti,ab,kw |
| #12 | MeSH descriptor: [Interviews as Topic] explode all trees |
| #13 | MeSH descriptor: [Focus Groups] explode all trees |
| #14 | MeSH descriptor: [Narration] explode all trees |
| #15 | MeSH descriptor: [Qualitative Research] explode all trees |
| #16 | (health professional* or doctor* or clinician* or consultant* or GP or general practitioner* or physician* or pharmacist*):kw,ti,ab |
| #17 | #10 or #11 or #12 or #13 or #14 or #15 or #16 |
| #18 | #9 and #17 |

### **WEB OF SCIENCE**

Indexes=SCI-EXPANDED, SSCI, CPCI-S, CPCI-SSH Timespan=All years

Date Run: 02/04/2021 Results: **662**

| #1 | TS=(rare disease* or rare diagnosis* or orphan disease*) |
| --- | --- |
| #2 | TS=(technology assessment) |
| #3 | TS=(NICE or "national institute for health and care excellence") |
| #4 | TS=(drug prescriptions) |
| #5 | TI=(guideline* or guidance or prescrib* or clinical protocol* or prescription*) |
| #6 | #2 OR #3 OR #4 OR #5 |
| #7 | #1 AND #6 |
| #8 | TS=(barrier* or facilit* or help* or hinder* or compliance or comply or complies or accept* or conform* or approv* or adhere* or strateg*) |
| #9 | TS=((("semi-structured" or semistructured or unstructured or informal or "in-depth" or indepth or "face-to-face" or structured or guide) NEAR/3 (interview* or discussion* or questionnaire*) ) or (focus group* or qualitative or ethnograph* or fieldwork or "field work" or "key informant") or (interviews as topic or focus groups or narration or qualitative research)) |
| #10 | TS=(health professional* or doctor* or clinician* or consultant* or GP or general practitioner* or physician* or pharmacist*) |
| #11 | #8 OR #9 OR #10 |
| #12 | #7 AND #11 |

**EMBASE**

Date Run: 08/04/2021 Results: **900**

| #1 | *"RARE DISEASES"/ |
| --- | --- |
| #2 | (rare disease* OR rare diagnos* OR orphan disease*).ti,ab |
| #3 | *(1 OR 2)* |
| #4 | *"BIOMEDICAL TECHNOLOGY ASSESSMENT"/ |
| #5 | (NICE OR "national institute for health and care excellence").ti,ab |
| #6 | *"PRESCRIPTION"/ |
| #7 | (guideline* OR guidance OR prescrib* OR clinical protocol* OR prescription).ti,ab |
| #8 | *(4 OR 5 OR 6 OR 7)* |
| #9 | *(3 AND 8)* |
| #10 | (barrier* OR facilit* OR help* OR hinder* OR compliance OR comply OR complies OR accept* OR conform* OR adhere OR strateg*).ti,ab |
| #11 | ((("semi-structured" OR semistructured OR unstructured OR informal OR "in-depth" OR in-depth OR "face-to-face" OR structured OR guide) ADJ3 (interview* OR discussion* OR questionnaire*)) OR (focus group* OR qualitative OR ethnography* OR fieldwork OR "field work" OR "key informant")).ti,ab |
| #12 | *INTERVIEW/ |
| #13 | *"QUALITATIVE RESEARCH"/ |
| #14 | *(10 OR 11 OR 12 OR 13)* |
| #15 | *(9 AND 14)* |

## **NICE specialised technology appraisal rare diseases search**

**NICE Health Technology Appraisals search**

| **Searches** | **Number of results** |
| --- | --- |
| Total results | 571 |
| Results on technologies | 171 |
| Excluding terminated | 142 |
| Rare diseases (non-oncology) | 29 |

### **MEDLINE**

Database: Ovid MEDLINE(R) and Epub Ahead of Print, In-Process, In-Data-Review & Other Non-Indexed Citations and Daily <1946 to March 18, 2021>

Date Run: 18/03/2021 Results: **1661**

| #1 | *Rare Diseases/ |
| --- | --- |
| #2 | (rare dis* or rare diagnos* or orphan dis*).mp. |
| #3 | *Hidradenitis Suppurativa/ |
| #4 | (acne inversa* or suppurativ* hidradeniti* or severe hidradeniti*).ti. |
| #5 | *Purpura, Thrombotic Thrombocytopenic/ |
| #6 | (thrombotic adj (thrombopenic or thrombocytopeni*) adj purpura).ti. |
| #7 | thrombotic microangiopathy.ti. |
| #8 | congenital microangiopathic hemolytic an?emia.ti. |
| #9 | mosch?owitz disease.ti. |
| #10 | ((schulman-upshaw or schulman upshaw or upshaw-schulman or upshaw schulman) adj syndrome).ti. |
| #11 | (upshaw factor and deficien*).ti,ab. |
| #12 | (hereditary adj (angioedema* or angioneurotic edema*)).ti. |
| #13 | (c1 esterase inhibitor deficien* or c1 inhibitor deficien*).ti. |
| #14 | *Primary Myelofibrosis/ |
| #15 | myelofibros*.ti. |
| #16 | *Anemia, Sickle Cell/ |
| #17 | (sickle cell adj (disease* or an?emia or disorder*)).ti. |
| #18 | (h?emoglobin s disease or hbs disease).ti. |
| #19 | (sickling disorder and h?emoglobin s).ti,ab. |
| #20 | *Myelodysplastic Syndromes/ |
| #21 | myelodysplastic syndrome*.ti. |
| #22 | dysmyelopoietic syndrome*.ti. |
| #23 | *Purpura, Thrombocytopenic, Idiopathic/ |
| #24 | autoimmune thrombocytopen*.ti. |
| #25 | werlhof* disease.ti. |
| #26 | idopathic thrombocytopenic purpura*.ti. |
| #27 | immune thrombocytopen*.ti. |
| #28 | *Liver Cirrhosis, Biliary/ |
| #29 | primary biliary cholangiti*.ti. |
| #30 | primary biliary cirrhosis.ti. |
| #31 | chylomicron?emia syndrome.mp. |
| #32 | *Gaucher Disease/ |
| #33 | acid beta-glucosidase deficiency.ti. |
| #34 | cerebroside lipidos*.ti,ab. |
| #35 | ((gba or glucocerebrosid*) adj deficien*).ti. |
| #36 | (gaucher* adj (disease or syndrome)).ti. |
| #37 | glucosylceramide.ti. |
| #38 | (kerasin adj (lipoidos* or thesaurismos*)).ti. |
| #39 | *Fabry Disease/ |
| #40 | (angiokeratoma adj1 diffus*).ti. |
| #41 | ((ceramide trihexosidase or gla or alpha-galactosidase a or alpha galactosidase a) adj deficiency).ti. |
| #42 | fabry* disease.ti. |
| #43 | hereditary dystopic lipidosis.ti. |
| #44 | *Mucopolysaccharidosis IV/ |
| #45 | ((mucopolysaccharidosis or mps) adj1 (IV* or 4*)).ti. |
| #46 | ((gains or galactosamine-6-sulfatase or galactosamine 6 sulfatase) adj deficien*).ti,ab. |
| #47 | (morquio adj1 (disease or syndrome)).ti. |
| #48 | (eccentro-osteochrondrodysplasia* or eccentro osteochrondrodysplasia* or eccentroosteochrondrodysplasia*).ti,ab. |
| #49 | *Epilepsies, Myoclonic/ |
| #50 | dravet syndrome*.ti. |
| #51 | ("severe myoclonic epilepsy of infancy" or "severe myoclonic epilepsy in infancy").ti. |
| #52 | severe polymorphic epilepsy of infancy.ti,ab. |
| #53 | ((borderland or borderline) adj SMEI).ti,ab. |
| #54 | "intractable childhood epilepsy with generalised tonic clonic seizures".ti,ab. |
| #55 | *Lennox Gastaut Syndrome/ |
| #56 | ((lennox-gastaut or lennox gastaut) adj syndrome*).ti. |
| #57 | *Neuronal Ceroid-Lipofuscinoses/ |
| #58 | (lipofuscinosis adj type 2).ti. |
| #59 | lipofuscinosis 2.ti. |
| #60 | *Muscular Atrophy, Spinal/ |
| #61 | spinal muscular atrophy.ti. |
| #62 | *Muscular Dystrophy, Duchenne/ |
| #63 | ((duchenne or pseudohypertrophic) adj1 muscular dystrophy).ti. |
| #64 | *Idiopathic Pulmonary Fibrosis/ |
| #65 | ((cryptogenic or idiopathic) adj fibrosing alveoliti*).ti. |
| #66 | fibrocystic pulmonary dysplasia.ti,ab. |
| #67 | idiopathic pulmonary fibros*.ti. |
| #68 | (fibrocystic disease adj1 pancrea*).ti. |
| #69 | mucoviscidosis.ti. |
| #70 | *Polycystic Kidney, Autosomal Dominant/ |
| #71 | adpkd.ti. |
| #72 | ((adult or type 2 or type ii) adj1 polycystic kidney disease).ti. |
| #73 | autosomal dominant polycystic kidney.ti. |
| #74 | *Amyloid Neuropathies, Familial/ |
| #75 | hereditary transthyretin amyloidosis.ti. |
| #76 | familial amyloid polyneuropathy.ti. |
| #77 | transthyretin-related hereditary amyloidosis.ti. |
| #78 | transthyretin amyloidosis.ti. |
| #79 | ATTR.ti,ab. |
| #80 | "Corino de Andrade's disease".ti. |
| #81 | *Cystitis, Interstitial/ |
| #82 | bladder pain syndrome*.ti. |
| #83 | interstitial cystiti*.ti. |
| #84 | painful bladder syndrome*.ti. |
| #85 | *Anti-Neutrophil Cytoplasmic Antibody-Associated Vasculitis/ |
| #86 | anca-associated vasculiti*or anca associated vasculiti*.ti,ab. |
| #87 | ((pauci-immune or pauci immune) adj vasculiti*).ti. |
| #88 | *Giant Cell Arteritis/ |
| #89 | (giant cell adj1 arteriti*).ti. |
| #90 | ((cranial or temporal) adj arteriti*).ti. |
| #91 | horton* disease.ti. |
| #92 | *Atypical Hemolytic Uremic Syndrome/ |
| #93 | (atypical hemolytic-uremic syndrome* or atypical hemolytic uremic syndrome*).ti. |
| #94 | ((non-shiga-like toxin-associated or non shiga like toxin associated or nonenteropathic or non stx or non-stx) adj hus*).ti,ab. |
| #95 | (*Uveitis/ or uveiti*.ti.) and (noninfectious or non-infectious).ti. |
| #96 | (kerasin adj1 histiocytos*).ti,ab. |
| #97 | or/1-96 |
| #98 | ((NICE or national institute for health) and care excellence).mp. |
| #99 | *Drug Prescriptions/ |
| #100 | (guideline* or guidance or prescrib* or clinical protocol* or prescription*).mp. or *Guideline Adherence/ or *Practice Guidelines as Topic/st [Standards] |
| #101 | 98 or 99 or 100 |
| #102 | 97 and 101 |
| #103 | (barrier* or facilit* or help* or hinder* or compliance or comply or complies or accept* or conform* or approv* or adhere* or strateg*).mp. |
| #104 | ((("semi-structured" or semistructured or unstructured or informal or "in-depth" or indepth or "face-to-face" or structured or guide) adj3 (interview* or discussion* or questionnaire*)) or (focus group* or qualitative or ethnograph* or fieldwork or "field work" or "key informant")).ti,ab. or interviews as topic/ or focus groups/ or narration/ or qualitative research/ |
| #105 | *"Attitude of Health Personnel"/ or *Practice Patterns, Physicians'/ |
| #106 | 103 or 104 or 105 |
| #107 | 102 and 106 |

### **COCHRANE**

Date Run: 02/04/21 Results: **737**

| #1 | MeSH descriptor: (1) explode all trees |
| --- | --- |
| #2 | (rare dis* or rare diagnos* or orphan dis*):ti,ab,kw |
| #3 | MeSH descriptor: [Hidradenitis Suppurativa] explode all trees |
| #4 | (acne inversa* or suppurativ* hidradeniti* or severe hidradeniti*):ti |
| #5 | MeSH descriptor: [Purpura, Thrombotic Thrombocytopenic] explode all trees |
| #6 | (thrombotic adj (thrombopenic or thrombocytopenia*) NEXT purpura):ti |
| #7 | (thrombotic microangiopathy):ti |
| #8 | (congenital microangiopathic hemolytic an?emia):ti |
| #9 | (mosch?owitz disease):ti |
| #10 | ((schulman-upshaw or schulman upshaw or upshaw-schulman or upshaw schulman) NEXTj syndrome):ti |
| #11 | (upshaw factor and deficien*):ti,ab |
| #12 | (hereditary NEXT (angioedema* or angioneurotic edema*)):ti |
| #13 | (c1 esterase inhibitor deficien* or c1 inhibitor deficien*):ti |
| #14 | MeSH descriptor: [Primary Myelofibrosis] explode all trees |
| #15 | (myelofibros*):ti |
| #16 | MeSH descriptor: [Anemia, Sickle Cell] explode all trees |
| #17 | (sickle cell NEXT (disease* or an?emia or disorder*)):ti |
| #18 | (?emoglobin s disease or hbs disease):ti |
| #19 | (sickling disorder and ?emoglobin s):ti,ab |
| #20 | MeSH descriptor: [Myelodysplastic Syndromes] explode all trees |
| #21 | (myelodysplastic syndrome*):ti |
| #22 | (dysmyelopoietic syndrome*):ti |
| #23 | MeSH descriptor: [Purpura, Thrombocytopenic, Idiopathic] explode all trees |
| #24 | (autoimmune thrombocytopen*):ti |
| #25 | (werlhof* disease):ti |
| #26 | (idiopathic thrombocytopenic purpura*):ti |
| #27 | (immune thrombocytopen*):ti |
| #28 | MeSH descriptor: [Liver Cirrhosis, Biliary] explode all trees |
| #29 | (primary biliary cholangiti*):ti |
| #30 | (primary biliary cirrhosis):ti |
| #31 | (chylomicron?emia syndrome):ti |
| #32 | MeSH descriptor: [Gaucher Disease] explode all trees |
| #33 | (acid beta-glucosidase deficiency):ti |
| #34 | (cerebroside lipidos*):ti,ab |
| #35 | ((gba or glucocerebrosid*) NEXT deficien*):ti |
| #36 | (gaucher* NEXT (disease or syndrome)):ti |
| #37 | (glycosylceramide):ti |
| #38 | (kerasin adj (lipoidos* or thesaurismos*)):ti |
| #39 | MeSH descriptor: [Fabry Disease] explode all trees |
| #40 | (angiokeratoma NEAR/1 diffus*):ti |
| #41 | ((ceramide trihexosidase or gal or alpha-galactosidase a or alpha galactosidase a) NEXT deficiency):ti |
| #42 | (fabry* disease):ti |
| #43 | (hereditary dystopic lipidosis):ti |
| #44 | MeSH descriptor: [Mucopolysaccharidosis IV] explode all trees |
| #45 | ((mucopolysaccharidosis or mps) NEAR/1 (IV* or 4*)):ti |
| #46 | ((gains or galactosamine-6 sulfatase or galactosamine 6 sulfastase) NEXT deficienc*):ti,ab |
| #47 | (morquio NEAR/1 (disease or syndrome)):ti |
| #48 | (eccentro-osteochrondrodysplasia* or eccentro osteochrondrodysplasia* or eccentroosteochrondrodysplasia*):ti,ab |
| #49 | MeSH descriptor: [Epilepsies, Myoclonic] explode all trees |
| #50 | (dravet syndrome*):ti |
| #51 | ("severe myoclonic epilepsy of infancy" or "severe myoclonic epilepsy in infancy"):ti |
| #52 | (severe polymorphic epilepsy of infancy):ti,ab |
| #53 | ((borderland or borderline) adj SMEI):ti,ab |
| #54 | ("intractable childhood epilepsy with generalised tonic clonic seizures"):ti,ab |
| #55 | MeSH descriptor: [Lennox Gastaut Syndrome] explode all trees |
| #56 | ((Lennox-gastaut or Lennox gastaut) NEXT syndrome*):ti |
| #57 | MeSH descriptor: [Neuronal Ceroid-Lipofuscinoses] explode all trees |
| #58 | (lipofuscinosis NEXT type 2):ti |
| #59 | (lipofuscinosis 2):ti |
| #60 | MeSH descriptor: [Muscular Atrophy, Spinal] explode all trees |
| #61 | (spinal muscular atrophy):ti |
| #62 | MeSH descriptor: [Muscular Dystrophy, Duchenne] explode all trees |
| #63 | ((Duchenne or pseudohypertrophic) NEAR/1 muscular dystrophy):ti |
| #64 | MeSH descriptor: [Idiopathic Pulmonary Fibrosis] explode all trees |
| #65 | ((cryptogenic or idiopathic) NEXT fibrosing alveoliti*):ti |
| #66 | (fibrocystic pulmonary dysplasia):ti,ab |
| #67 | (idiopathic pulmonary fibros*):ti |
| #68 | (fibrocystic disease adj1 pancrea*):ti |
| #69 | (mucoviscidosis):ti |
| #70 | MeSH descriptor: [Polycystic Kidney, Autosomal Dominant] explode all trees |
| #71 | (adpkd):ti |
| #72 | ((adult or type 2 or type ii) NEAR/1 polycystic kidney disease):ti |
| #73 | (autosomal dominant polycystic kidney):ti |
| #74 | MeSH descriptor: [Amyloid Neuropathies, Familial] explode all trees |
| #75 | (hereditary transthyretin amyloidosis):ti |
| #76 | (familial amyloid polyneuropathy):ti |
| #77 | (transthyretin-related hereditary amyloidosis):ti |
| #78 | (transthyretin amyloidosis):ti |
| #79 | (ATTR):ti,ab |
| #80 | ("Corino de Andrade's disease"):ti |
| #81 | MeSH descriptor: [Cystitis, Interstitial] explode all trees |
| #82 | (bladder pain syndrome*):ti |
| #83 | (interstitial cystiti*):ti |
| #84 | (painful bladder syndrome*):ti |
| #85 | MeSH descriptor: [Anti-Neutrophil Cytoplasmic Antibody-Associated Vasculitis] explode all trees |
| #86 | (anca-associated vasculiti* or anca associated vasculiti*):ti,ab |
| #87 | ((pauci-immune or pauci immune) adj vasculiti*):ti |
| #88 | MeSH descriptor: [Giant Cell Arteritis] explode all trees |
| #89 | (giant cell adj1 arteriti*):ti |
| #90 | ((cranial or temporal) adj arteriti*):ti |
| #91 | (horton* disease):ti |
| #92 | MeSH descriptor: [Atypical Hemolytic Uremic Syndrome] explode all trees |
| #93 | (atypical hemolytic-uremic syndrome* or atypical hemolytic uremic syndrome):ti |
| #94 | ((non-shiga-like toxin-associated or non shiga like toxin associated or nonenteropathic or non stx or non-stx) NEXT hus*):ti,ab |
| #95 | (uveiti*):ti and (noninfectious or non-infectious):ti |
| #96 | MeSH descriptor: [Uveitis] explode all trees |
| #97 | (kerasin NEAR/1 histiocytos*):ti,ab |
| #98 | (OR #1-#97) |
| #99 | ((NICE or national institute for health) and care excellence):kw,ti,ab |
| #100 | MeSH descriptor: [Drug Prescriptions] explode all trees |
| #101 | (guideline* or guidance or prescrib* or clinical protocol* or prescription*):kw |
| #102 | MeSH descriptor: [Guideline Adherence] explode all trees |
| #103 | MeSH descriptor: [Practice Guidelines as Topic] explode all trees |
| #104 | #99 or #100 or #101 or #102 or #103 |
| #105 | #98 and #104 |
| #106 | (barrier* or facilit* or help* or hinder* or compliance or comply or complies or accept* or conform* or approv* or adhere* or strateg*):kw,ti,ab |
| #107 | ((("semi-structured" or semistructured or unstructured or informal or "in-depth" or indepth or "face-to-face" or structured or guide) NEAR/3 (interview* or discussion* or questionnaire*)) or (focus group* or qualitative or ethnograph* or fieldwork or "field work" or "key informant")):ti,ab |
| #108 | MeSH descriptor: [Interviews as Topic] explode all trees |
| #109 | MeSH descriptor: [Focus Groups] explode all trees |
| #110 | MeSH descriptor: [Narration] explode all trees |
| #111 | MeSH descriptor: [Qualitative Research] explode all trees |
| #112 | MeSH descriptor: [Attitude of Health Personnel] explode all trees |
| #113 | MeSH descriptor: [Practice Patterns, Physicians'] explode all trees |
| #114 | #106 or #107 or #108 or #109 or #110 or #111 or #112 or #113 |
| #115 | #105 and #114 |

### **Web of Science**

Indexes=SCI-EXPANDED, SSCI, CPCI-S, CPCI-SSH Timespan=All years

Date Run: 02/04/2021 Results: **650**

| #1 | TI=(rare dis* or rare diagnos* or orphan dis*) |
| --- | --- |
| #2 | TI=(hidradenitis suppurativa) |
| #3 | ti=(acne inversa* or suppurativ* hidradeniti* or severe hidradeniti*) |
| #4 | TI=(thrombotic NEAR (thrombopenic or thrombocytopeni*) NEAR purpura) |
| #5 | TI=(thrombotic microangiopathy) |
| #6 | TI=(congenital microangiopathic h?emolytic an?emia) |
| #7 | TI=(mosch?owitz disease) |
| #8 | TI=((schulman-upshaw or Schulman upshaw or upshaw-schulman or upshaw schulman) NEXT syndrome) |
| #9 | TI=(upshaw factor and deficien*) |
| #10 | TI=(hereditary NEXT (angioedema* or angioneurotic edema*) ) |
| #11 | TI=(c1 esterase inhibitor deficien* or c1 inhibitor deficien*) |
| #12 | TI=(primary myelofibrosis) |
| #13 | TI=(myelofibros*) |
| #14 | TI=(sickle cell NEAR (disease* or an?emia or disorder) ) |
| #15 | TI=(h?emoglobin s disease or hbs disease) |
| #16 | TI=(sickling disorder and h?emoglobin s) |
| #17 | TI=(myelodysplastic syndrome) |
| #18 | TI=(dysmyelopoietic syndrome) |
| #19 | TI=(idiopathic thrombocytopenic purpura) |
| #20 | TI=(autoimmune thrombocytopen*) |
| #21 | TI=(werlhof* disease) |
| #22 | TI=(immune thrombocytopen*) |
| #23 | TI=(primary biliary cirrhosis) |
| #24 | TI=(primary biliary cholangitis) |
| #25 | TI=(chylomicron?emia syndrome) |
| #26 | TI=(gaucher disease) |
| #27 | TI=(acid beta-glucosidase deficiency) |
| #28 | TI=(cerebroside lipidos*) |
| #29 | TI=((gba or glucocerebrosid*) NEAR deficien*) |
| #30 | TI=(gaucher syndrome) |
| #31 | TI=(glucosylceramide) |
| #32 | TI=(kerasin NEAR (lipoidos* or thesaurismos*) ) |
| #33 | TI=(fabry disease) |
| #34 | TI=(angiokeratoma NEAR diffus*) |
| #35 | TI=((ceramide trihexosidase or gal or alpha-galactosidase a or alpha galactosidase) NEXT deficiency) |
| #36 | TI=(hereditary dystopic lipidosis) |
| #37 | TI=((mucopolysaccharidosis IV) or (mucopolysaccharidosis 4) ) |
| #38 | TI=((gains or galactosamine-6-sulfatase or galactosamine 6 sulfatase) NEXT deficien*) |
| #39 | TI=(morquio NEAR (disease or syndrome) ) |
| #40 | TI=(eccentro-osteochrondrodysplasia* or eccentro osteochrondrodysplasia* or eccentroosteochrondrodysplasia*) |
| #41 | TI=(myoclonic epileps*) |
| #42 | TI=(dravet syndrome*) |
| #43 | TI=("severe myoclonic epilepsy of infancy" or "severe myoclonic epilepsy in infancy") |
| #44 | TI=(severe polymorphic epilepsy of infancy) |
| #45 | TI=((borderland or borderline) NEAR SMEI) |
| #46 | TI=("intractable childhood epilepsy with generalised tonic clonic seizures") |
| #47 | TI=((Lennox gastaut or Lennox-gastaut) NEXT syndrome) |
| #48 | TI=(neuronal ceroid-lipofuscinoses) |
| #49 | TI=((lipofuscinosis 2) or (lipofuscinosis type 2) ) |
| #50 | TI=(spinal muscular atrophy) |
| #51 | TI=(Duchenne muscular dystrophy) |
| #52 | TI=((Duchenne or pseudohypertrophic) NEAR muscular dystrophy) |
| #53 | TI=(idiopathic pulmonary fibros*) |
| #54 | TI=((cryptogenic or idiopathic) NEAR fibrosing alveoliti*) |
| #55 | TI=(fibrocystic pulmonary dysplasia) |
| #56 | TI=(fibrocystic disease NEAR pancrea*) |
| #57 | TI=(mucoviscidosis) |
| #58 | TI=(autosomal dominant polycystic kidney disease) |
| #59 | TI=(adpkd) |
| #60 | TI=((adult polycystic kidney disease) or (type 2 polycystic kidney disease) or (type II polycystic kidney disease) ) |
| #61 | TI=(familial amyloid neuropath*) |
| #62 | TI=(hereditary transthyretin amyloidosis) |
| #63 | TI=(familial amyloid polyneuropathy) |
| #64 | TI=((transthyretin-related hereditary amyloidosis) or (transthyretin amyloidosis) ) |
| #65 | TI=(ATTR) |
| #66 | TI=("Corino de Andrade's disease") |
| #67 | TI=(interstitial cystiti*) |
| #68 | TI=(bladder pain syndrome*) |
| #69 | TI=(painful bladder syndrome) |
| #70 | TI=(anti-neutrophil cytoplasmic antibody-associated vasculitis) |
| #71 | TI=(anca-associated vasculiti* or anca associated vasculiti*) |
| #72 | TI=((pauci-immune or pauci immune) NEXT vasculiti*) |
| #73 | TI=(giant cell arteriti*) |
| #74 | TI=((cranial or temporal) NEAR arteriti*) |
| #75 | TI=(horton* disease) |
| #76 | TI=(atypical h?emolytic ur?emic syndrome) |
| #77 | TI=(atypical h?emolytic-ur?emic syndrome* or atypical h?emolytic ur?emic syndrome*) |
| #78 | TI=((non-shiga-like toxin-associated or non shiga like toxin associated or nonenteropathic or non stx or non-stx) NEXT HUS*) |
| #79 | TI=(uveiti* and (non-infectious or noninfectious) ) |
| #80 | TI=(kerasin NEAR histiocytos*) |
| #81 | #80 OR #79 OR #78 OR #77 OR #76 OR #75 OR #74 OR #73 OR #72 OR #71 OR #70 OR #69 OR #68 OR #67 OR #66 OR #65 OR #64 OR #63 OR #62 OR #61 OR #60 OR #59 OR #58 OR #57 OR #56 OR #55 OR #54 OR #53 OR #52 OR #51 OR #50 OR #49 OR #48 OR #47 OR #46 OR #45 OR #44 OR #43 OR #42 OR #41 OR #40 OR #39 OR #38 OR #37 OR #36 OR #35 OR #34 OR #33 OR #32 OR #31 OR #30 OR #29 OR #28 OR #27 OR #26 OR #25 OR #24 OR #23 OR #22 OR #21 OR #20 OR #19 OR #18 OR #17 OR #16 OR #15 OR #14 OR #13 OR #12 OR #11 OR #10 OR #9 OR #8 OR #7 OR #6 OR #5 OR #4 OR #3 OR #2 OR #1 |
| #82 | TI=((NICE or national institute for health) and care excellence) |
| #83 | TS=(guideline* or guidance or prescrib* or clinical protocol* or prescription*) |
| #84 | #83 OR #82 |
| #85 | #84 AND #81 |
| #86 | TI=(barrier* or facilit* or compliance or comply or complies or accept* or conform* or adhere* or approv* or strateg*) |
| #87 | TS=("semi-structured" or semistructured or unstructured or informal or "in-depth" or indepth or "face-to-face" or structured or guide or interview* or discussion* or questionnaire* or focus group* or qualitative or ethnograph* or fieldwork or "field work" or "key informant") |
| #88 | #87 OR #86 |
| #89 | #88 AND #85 |

### **EMBASE**

Date Run: 02/04/21 Results: **1071**

| #1 | *"RARE DISEASE"/ |
| --- | --- |
| #2 | (rare dis* OR rare diagnos* OR orphan dis*).ti |
| #3 | *"SUPPURATIVE HIDRADENITIS"/[View Results (3,711)](https://hdas.nice.org.uk/strategy/1005128/48/#show-results) |
| #4 | (acne inversa* OR suppurativ* hidradeniti* OR severe hidradeniti*).ti |
| #5 | *"THROMBOTIC THROMBOCYTOPENIC PURPURA"/ |
| #6 | ((thrombotic ADJ (thrombopenic OR thrombocytopeni*)) ADJ purpura).ti |
| #7 | (thrombotic microangiopathy).ti |
| #8 | (congenital microangiopathic hemolytic an?emia).ti |
| #9 | (mosch?owitz disease).ti |
| #10 | ((schulman-upshaw OR schulman upshaw OR upshaw-schulman OR upshaw schulman) ADJ syndrome).ti |
| #11 | (upshaw factor AND deficien*).ti,ab |
| #12 | (hereditary ADJ (angioedema* OR angioneurotic edema*)).ti |
| #13 | (c1 esterase inhibitor deficien* OR c1 inhibitor deficien*).ti |
| #14 | *"MYELOFIBROSIS"/ |
| #15 | (myelofibros*).ti |
| #16 | *"SICKLE CELL ANAEMIA"/ |
| #17 | (sickle cell ADJ (disease* OR an?emia OR disorder*)).ti |
| #18 | (h?emoglobin s disease OR hbs disease).ti |
| #19 | (sickling disorder AND h?emoglobin s).ti,ab |
| #20 | *"MYELODYSPLASTIC SYNDROME"/ |
| #21 | (myelodysplastic syndrome*).ti |
| #22 | (dysmyelopoietic syndrome*).ti |
| #23 | *" IDIOPATHIC THROMBOCYTOPENIC PURPURA"/ |
| #24 | (autoimmune thrombocytopen*).ti |
| #25 | (werlhof* disease).ti |
| #26 | (idopathic thrombocytopenic purpura*).ti |
| #27 | (immune thrombocytopen*).ti |
| #28 | *"BILIARY CIRRHOSIS"/ |
| #29 | (primary biliary cholangiti*).ti |
| #30 | (primary biliary cirrhosis).ti |
| #31 | (chylomicron?emia syndrome).ti,ab |
| #32 | *"GAUCHER DISEASE"/ |
| #33 | (acid beta-glucosidase deficien*).ti |
| #34 | (cerebroside lipidos*).ti,ab |
| #35 | ((gba OR glucocerebrosid*) ADJ deficien*).ti |
| #36 | (gaucher* ADJ (disease OR syndrome)).ti |
| #37 | (glucosylceramide).ti |
| #38 | (kerasin ADJ (lipoidos* OR thesaurismos*)).ti |
| #39 | *"FABRY DISEASE"/ |
| #40 | (angiokeratoma ADJ1 diffus*).ti |
| #41 | ((ceramide trihexosidase OR gla OR alpha-galactosidase a OR alpha galactosidase a) ADJ deficien*).ti |
| #42 | (fabry* disease).ti |
| #43 | (hereditary dystopic lipidosis).ti |
| #44 | *"MUCOPOLYSACCHARIDOSIS"/ |
| #45 | ((mucopolysaccharidosis OR mps) ADJ1 (IV* OR 4*)).ti |
| #46 | ((gains OR galactosamine-6-sulfatase OR galactosamine 6 sulfatase) ADJ deficien*).ti,ab |
| #47 | (morquio ADJ1 (disease OR syndrome)).ti |
| #48 | (eccentro-osteochrondrodysplasia* OR eccentro osteochrondrodysplasia* OR eccentroosteochrondrodysplasia*).ti,ab |
| #49 | *" MYOCLONUS EPILEPSY"/ |
| #50 | (dravet syndrome*).ti |
| #51 | ("severe myoclonic epilepsy of infancy" OR "severe myoclonic epilepsy in infancy").ti |
| #52 | (severe polymorphic epilepsy of infancy).ti,ab |
| #53 | ((borderland OR borderline) ADJ SMEI).ti,ab |
| #54 | ("intractable childhood epilepsy with generalised tonic clonic seizures").ti,ab |
| #55 | *"LENNOX GASTAUT SYNDROME"/ |
| #56 | ((lennox-gastaut OR lennox gastaut) ADJ syndrome*).ti |
| #57 | *"NEURONAL CEROID LIPOFUSCINOSIS"/ |
| #58 | (lipofuscinosis ADJ type 2).ti |
| #59 | (lipofuscinosis 2).ti |
| #60 | *" SPINAL MUSCULAR ATROPHY"/ |
| #61 | (spinal muscular atrophy).ti |
| #62 | *"DUCHENNE MUSCULAR DYSTROPHY"/ |
| #63 | ((duchenne OR pseudohypertrophic) ADJ1 muscular dystrophy).ti |
| #64 | *"IDIOPATHIC PULMONARY FIBROSIS"/ |
| #65 | ((cryptogenic OR idiopathic) ADJ fibrosing alveoliti*).ti |
| #66 | (fibrocystic pulmonary dysplasia).ti,ab |
| #67 | (idiopathic pulmonary fibros*).ti |
| #68 | (fibrocystic disease ADJ1 pancrea*).ti |
| #69 | (mucoviscidosis).ti |
| #70 | *"KIDNEY POLYCYSTIC DISEASE"/ |
| #71 | (adpkd).ti |
| #72 | ((adult OR type 2 OR type ii) ADJ1 polycystic kidney disease).ti |
| #73 | (autosomal dominant polycystic kidney).ti |
| #74 | *" FAMILIAL AMYLOID POLYNEUROPATHY"/ |
| #75 | (hereditary transthyretin amyloidosis).ti |
| #76 | (familial amyloid polyneuropathy).ti |
| #77 | (transthyretin-related herary amyloidosis).ti |
| #78 | (transthyretin amyloidosis).ti |
| #79 | (ATTR).ti |
| #80 | ("Corino de Andrade's disease").ti |
| #81 | *"INTERSTITIAL CYSTITIS"/ |
| #82 | (bladder pain syndrome*).ti |
| #83 | (interstitial cystiti*).ti |
| #84 | (painful bladder syndrome*).ti |
| #85 | *"ANCA ASSOCIATED VASCULITIS"/ |
| #86 | (anca-associated vasculiti*or anca associated vasculiti*).ti,ab |
| #87 | ((pauci-immune OR pauci immune) ADJ vasculiti*).ti |
| #88 | *"GIANT CELL ARTERITIS"/ |
| #89 | (giant cell ADJ1 arteriti*).ti |
| #90 | ((cranial OR temporal) ADJ arteriti*).ti |
| #91 | (horton* disease).ti |
| #92 | *"ATYPICAL HEMOLYTIC UREMIC SYNDROME"/ |
| #93 | (atypical hemolytic-uremic syndrome* OR atypical hemolytic uremic syndrome*).ti |
| #94 | ((non-shiga-like toxin-associated OR non shiga like toxin associated OR nonenteropathic OR non stx OR non-stx) ADJ hus*).ti,ab |
| #95 | (*"UVEITIS"/ OR uveiti*) AND (noninfectious OR non-infectious) |
| #96 | (kerasin ADJ1 histiocytos*).ti,ab |
| #97 | *(1 OR 2 OR 3 OR 4 OR 5 OR 6 OR 7 OR 8 OR 9 OR 10 OR 11 OR 12 OR 13 OR 14 OR 15 OR 16 OR 17 OR 18 OR 19 OR 20 OR 21 OR 22 OR 23 OR 24 OR 25 OR 26 OR 27 OR 28 OR 29 OR 30 OR 31 OR 32 OR 33 OR 34 OR 35 OR 36 OR 37 OR 38 OR 39 OR 40 OR 41 OR 42 OR 43 OR 44 OR 45 OR 46 OR 47 OR 48 OR 49 OR 50 OR 51 OR 52 OR 53 OR 54 OR 55 OR 56 OR 57 OR 58 OR 59 OR 60 OR 61 OR 62 OR 63 OR 64 OR 65 OR 66 OR 67 OR 68 OR 69 OR 70 OR 71 OR 72 OR 73 OR 74 OR 75 OR 76 OR 77 OR 78 OR 79 OR 80 OR 81 OR 82 OR 83 OR 84 OR 85 OR 86 OR 87 OR 88 OR 89 OR 90 OR 91 OR 92 OR 93 OR 94 OR 95 OR 96)* |
| #98 | ((NICE OR national institute for health) AND care excellence).ti,ab |
| #99 | *"PRESCRIPTION"/ |
| #100 | guideline* OR guidance OR prescrib* OR clinical protocol* OR prescription* OR *"PROTOCOL COMPLIANCE"/ OR *"PRACTICE GUIDELINE"/ |
| #101 | *(98 OR 99 OR 100)* |
| #102 | *(97 AND 101)* |
| #103 | (barrier* OR faciliat* OR compliance OR comply OR complies OR accept* OR conform* OR adhere*).ti,ab |
| #104 | (("semi-structured" OR semistructured OR unstructured OR informal OR "in-depth" OR indepth OR "face-to-face" OR structured OR guide) ADJ3 (interview* OR discussion* OR questionnaire*)) OR (focus group* OR qualitative OR ethnograph* OR fieldwork OR "field work" OR "key informant") OR *"INTERVIEW"/ OR *"QUALITATIVE RESEARCH"/ |
| #105 | *"HEALTH PERSONNEL ATTITUDE"/ OR *"CLINICAL PRACTICE"/ |
| #106 | *(103 OR 104 OR 105)* |
| #107 | *(102 AND 107)* |

## **Orphanet Journal hand-search**

Orphanet Journals 12 (2017) to 16 (2021)

| Studies assessed at title stage | 1173 |
| --- | --- |
| Studies assessed at title stage (duplicates removed) | 1173 |
| Studies assessed at abstract stage | 7 |
| Studies assessed at full-text stage | 7 |
| Studies included in systematic review | 0 |

## **Pearl-growing subject search**

Search date: 28/02/2021

## **Grey Literature provided by the NICE Health Technology Adoption team**

| Documents provided | 5 |
| --- | --- |
| Studies assessed at full-text stage | 5 |
| Studies assessed at full-text stage (duplicates removed) | 5 |
| Studies included in systematic review | 0 |

NICE Technology appraisal guidance (TA388): Sacubitril valsartan for treating symptomatic chronic heart failure with reduced ejection fraction

NICE Medical technology consultation (MT413): Supporting documentation – Rezum

NICE Medical technology consultation (MT417): Supporting documentation – Axonics

NICE Adoption report (DG12 & NG80): Measuring fractional exhaled nitric oxide concentration in asthma

NICE Adoption report: RetinaScan
