# Supplementary figures and images for: Barriers and facilitators to the implementation of guidelines in rare diseases: a systematic review
Source: Orphanet J Rare Dis. 2023 Jun 7;18:140. doi: 10.1186/s13023-023-02667-9 (PMC10246545; doi:10.1186/s13023-023-02667-9)

## **Additional file 3 – Search strategy**

##
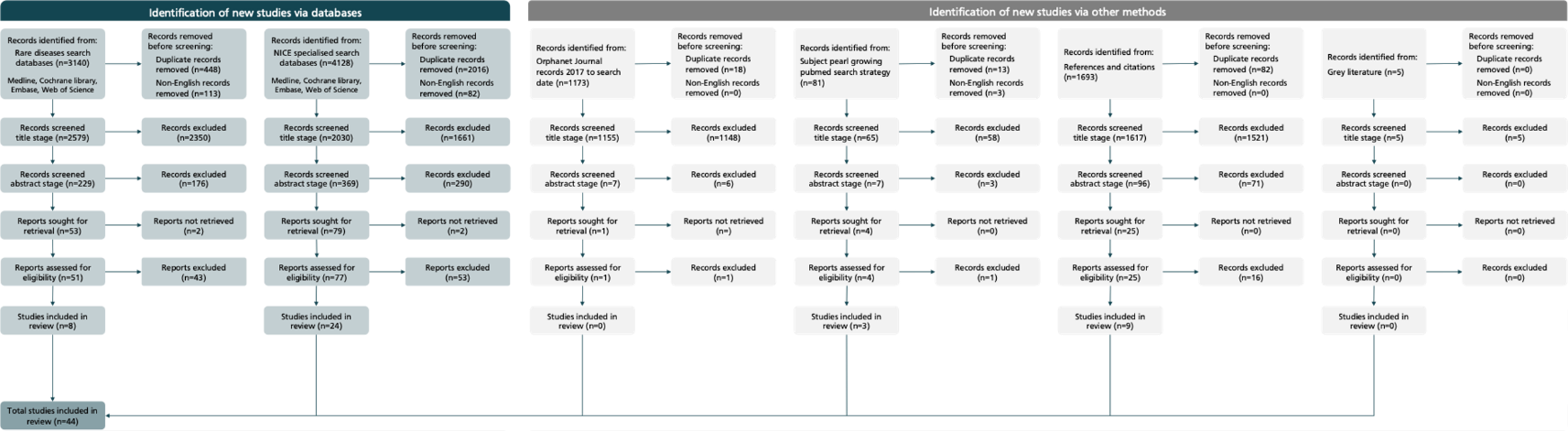

Supplement: Supplementary file 3 — Additional file 3. PRISMA flow diagram. [file 13023_2023_2667_MOESM3_ESM.docx]

## **Additional file 8 – Granular infographic of determinants of practice distribution**

## **
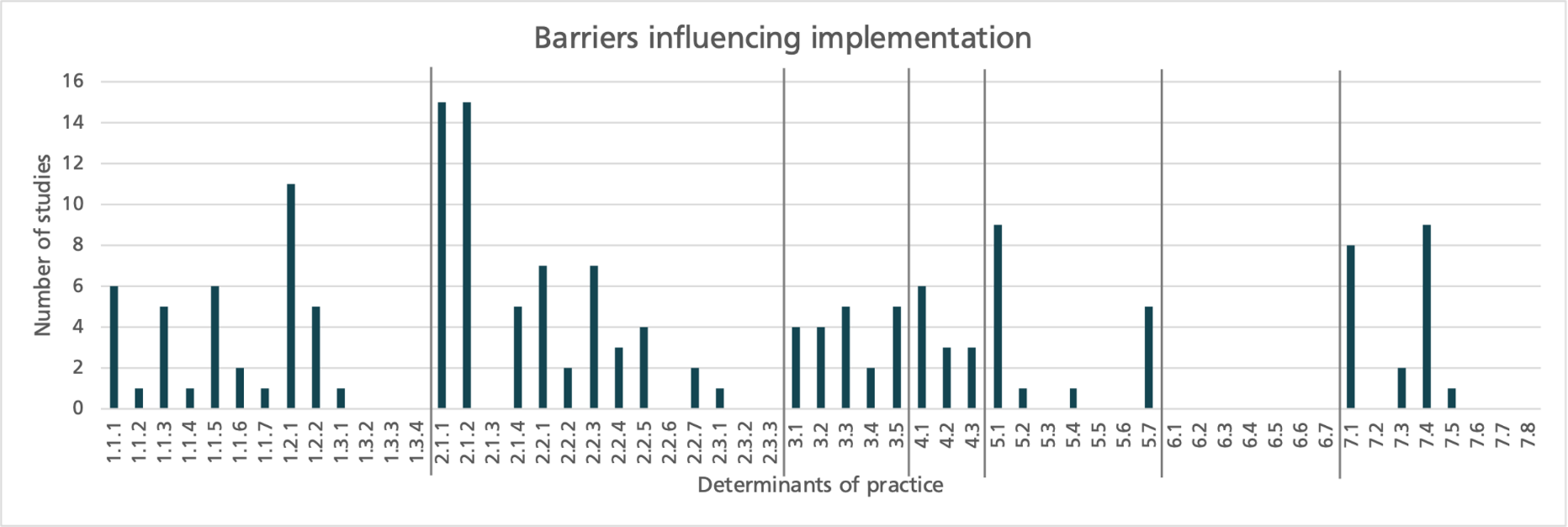
**

**
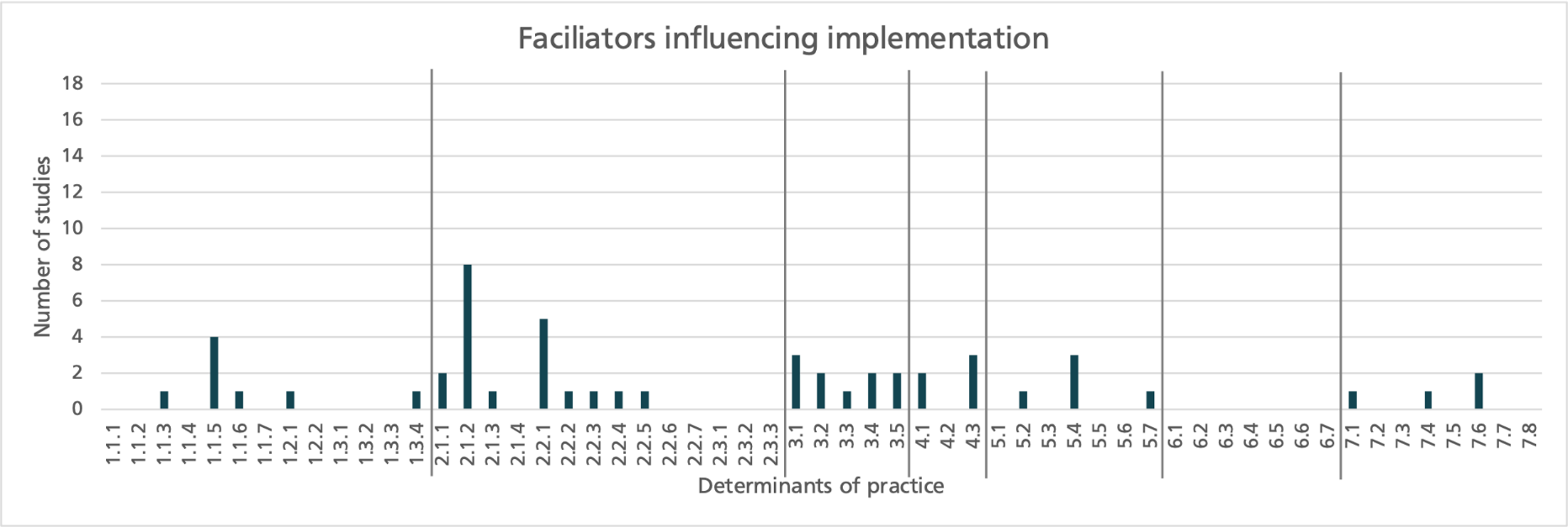
**

Supplement: Supplementary file 8 — Additional file. 8 Granular infographic of determinants of practice distribution. [file 13023_2023_2667_MOESM8_ESM.docx]
