# Supplementary material for: Barriers and facilitators to the implementation of guidelines in rare diseases: a systematic review
Source: Orphanet J Rare Dis. 2023 Jun 7;18:140. doi: 10.1186/s13023-023-02667-9 (PMC10246545; doi:10.1186/s13023-023-02667-9)
Supplement: Supplementary file 4 — Additional file 4. List of excluded studies at full-text stage. [file 13023_2023_2667_MOESM4_ESM.docx]

## **Additional file 4 – List of excluded studies at full-text stage**

| **Rare diseases search** | | |
| --- | --- | --- |
| **Authors** | **Database ID** | **Reason for exclusion** |
| McCabe et al | 8606477 | No established guideline or consensus document |
| Reshef et al | 19160940 | Review of treatment |
| Krug et al | 19407439 | Descriptive (no barriers or facilitators) |
| Griffith et al | 20004776 | No established guideline or consensus document |
| Fioredda et al | 21448998 | Guideline development |
| Bernstein & Moellman | 22691903 | Descriptive (no barriers or facilitators) |
| Wise | 23045352 | Review of treatment |
| Matzdorff et al | 23664509 | Review of treatment |
| Cicardi et al | 23689238 | Review of treatment |
| Noh & Lee | 24612142 | Descriptive (no barriers or facilitators) |
| van Karnebeek et al | 24748525 | Guideline development |
| Ampuero et al | 25075657 | No established guideline or consensus document |
| Bellutti Enders et al | 27515057 | Guideline development |
| Lupa & Wise | 27841771 | Review of treatment |
| Khodyakov et al | 28455279 | Guideline development |
| Stoller | 29325986 | Review of treatment |
| Torrent-Farnell et al | 29572017 | Guideline development |
| Li et al | 29909591 | Descriptive (no barriers or facilitators) |
| Crowley et al | 30021461 | Descriptive (no barriers or facilitators) |
| Reimer et al | 30409176 | No established guideline or consensus document |
| Bourdoncle et al | 30777102 | Review of treatment |
| Bachoud-Levi et al | 31333565 | Guideline development |
| Heard et al | 31907071 | No established guideline or consensus document |
| Torregrosa et al | 32236878 | Descriptive (no barriers or facilitators) |
| Kam & Ruiz | 33002306 | Descriptive (no barriers or facilitators) |
| Kjellstrom et al | 33456751 | Descriptive (no barriers or facilitators) |
| Baumgartner et al | 33681659 | No established guideline or consensus document |
| Strzelczyk et al | 33792454 | Descriptive (no barriers or facilitators) |
| Heringer et al | 70441898 | No established guideline or consensus document |
| Hundsberger et al | 70749557 | No established guideline or consensus document |
| van der Zeijden & Huizer | 71238280 | Conference abstract |
| Cannizzo et al | 626071036 | Review of treatment |
| Bertelsen et al | 20865287 | Descriptive (no barriers or facilitators) |
| Leblizq et al | 21035819 | Descriptive (no barriers or facilitators) |
| Saint-Raymond et al | WOS:000338206500001 | Review of treatment |
| Jose et al | 27129449 | Review of treatment |
| Mincarone et al | 28359063 | No established guideline or consensus document |
| Schuller et al | 29455022 | Review of treatment |
| Malinowski et al | 30483124 | No established guideline or consensus document |
| Norman et al | 31407784 | Review of treatment |
| Meyer et al | 30886730 | Descriptive (no barriers or facilitators) |
| Bouwman et al | WOS:000528546500020 | No established guideline or consensus document |
| Vazquez et al | 32671468 | Review of treatment |
|  |  |  |
| **NICE specialised technology appraisal search** | | |
| **Authors** | **Database ID** | **Reason for exclusion** |
| Marks et al | 15670225 | Descriptive (no barriers or facilitators) |
| Weinreb et al | 16247743 | Guideline development |
| Linthorst et al | 16249196 | Descriptive (no barriers or facilitators) |
| Smith et al | 16651336 | Descriptive (no barriers or facilitators) |
| Charles et al | 16999718 | Descriptive (no barriers or facilitators) |
| Gernsheimer & McCrae | 17934366 | Review of treatment |
| Cousins et al | 18563007 | Descriptive (no barriers or facilitators) |
| McMillan et al | 20437929 | Descriptive (no barriers or facilitators) |
| Riedl | 22386830 | Review of treatment |
| Dispenza & Craig | 22584193 | Descriptive (no barriers or facilitators) |
| Qureshi et al | 22944622 | Descriptive (no barriers or facilitators) |
| Michel | 23664517 | Review of treatment |
| Lozano et al | 27098812 | Descriptive (no barriers or facilitators) |
| Cottin et al | 27730153 | Descriptive (no barriers or facilitators) |
| Goddard | 28862731 | Review of treatment |
| Landfeldt | 29198861 | Non-medication related |
| Cherrez-Ojeda et al | 29321018 | Descriptive (no barriers or facilitators) |
| Temsah et al | 29455226 | No established guideline or consensus document |
| Smith et al | 30314826 | No established guideline or consensus document |
| Jabour et al | 31410784 | Review of treatment |
| El-Amin et al | 31817043 | No established guideline or consensus document |
| Turktas et al | 32163363 | Descriptive (no barriers or facilitators) |
| Chanias et al | 33371225 | Descriptive (no barriers or facilitators) |
| Dispenza & Craig | 70669785 | Descriptive (no barriers or facilitators) |
| Lu et al | 70963206 | Descriptive (no barriers or facilitators) |
| Hernandez-Perez et al | 71697297 | Review of treatment |
| Reddy & Craig | 71789592 | Descriptive (no barriers or facilitators) |
| Reilly et al | 71802912 | Descriptive (no barriers or facilitators) |
| Oltmanns et al | 71841070 | Review of treatment |
| Schlenz et al | 72173046 | Non-medication related |
| Miura & Azuma | 604096424 | Review of treatment |
| Ingram & McPhee | 25913663 | Descriptive (no barriers or facilitators) |
| McGrath et a | 619317868 | Descriptive (no barriers or facilitators) |
| Washko et al | 621728573 | Non-medication related |
| Harrison et al | 627190520 | Descriptive (no barriers or facilitators) |
| Westerman et al | 633023143 | Descriptive (no barriers or facilitators) |
| Shaman et al | 633023360 | Conference abstract |
| Karkoska et al | 634309552 | Descriptive (no barriers or facilitators) |
| Sivakumar et al | 634455434 | Descriptive (no barriers or facilitators) |
| Howard et al | 2004593148 | Descriptive (no barriers or facilitators) |
| Gilbert et al | 2004767908 | Review of treatment |
| Fidler et al | 2005877442 | Descriptive (no barriers or facilitators) |
| Schwartz et al | 2005912878 | Descriptive (no barriers or facilitators) |
| Pinto et al | 2008436140 | Descriptive (no barriers or facilitators) |
| Villa-Forte & Hoffman | 18446138 | Descriptive (no barriers or facilitators) |
| Cottin et al | 24361163 | Descriptive (no barriers or facilitators) |
| Reeves et al | 25320061 | Non-medication related |
| Bollinger et al | 20842753 | Non-medication related |
| Benson et al | 22170631 | Non-medication related |
| Routledge et al | WOS:000334923300244 | Guideline development |
| De Andrade et al | WOS:000466776700088 | Descriptive (no barriers or facilitators) |
| Howard et al | WOS:000510805200192 | Descriptive (no barriers or facilitators) |
| Schwartz et al | WOS:000540349504447 | Descriptive (no barriers or facilitators) |
|  |  |  |
| **Orphanet Journal hand-search** | | |
| **Authors** | **Database ID** | **Reason for exclusion** |
| Wegberg et al | 29025426 | Guideline development |
|  |  |  |
| **Pearl-growing subject search** | | |
| **Authors** | **Database ID** | **Reason for exclusion** |
| Singh et al | 27797455 | Review of treatment |
|  |  |  |
| **References & Citations** | | |
| **Authors** | **Database ID** | **Reason for exclusion** |
| Cicardi et al | 2013670 | Review of treatment |
| Cicardi & Zingale | 14572813 | Descriptive (no barriers or facilitators) |
| Frank et al | 17085283 | Descriptive (no barriers or facilitators) |
| Moran et al | 21115772 | Guideline development |
| Banerji et al | 25976438 | Descriptive (no barriers or facilitators) |
| Oskoui et al | 27685758 | No established guideline or consensus document |
| Brennan-Cook | 29846351 | Descriptive (no barriers or facilitators) |
| Smith et al | 30402270 | Descriptive (no barriers or facilitators) |
| Tamirou et al | 30564454 | Descriptive (no barriers or facilitators) |
| Terzioglu et al | 36452014 | Descriptive (no barriers or facilitators) |
| Pandya et al | [1526-632X](http://www.worldcat.org/issn/1526-632X) | Descriptive (no barriers or facilitators) |
