# Supplementary material for: Barriers and facilitators to the implementation of guidelines in rare diseases: a systematic review
Source: Orphanet J Rare Dis. 2023 Jun 7;18:140. doi: 10.1186/s13023-023-02667-9 (PMC10246545; doi:10.1186/s13023-023-02667-9)
Supplement: Supplementary file 5 — Additional file 5. Included study characteristics. [file 13023_2023_2667_MOESM5_ESM.docx]

## **Additional file 5 – Included study characteristics**
