# Supplementary material for: Barriers and facilitators to the implementation of guidelines in rare diseases: a systematic review
Source: Orphanet J Rare Dis. 2023 Jun 7;18:140. doi: 10.1186/s13023-023-02667-9 (PMC10246545; doi:10.1186/s13023-023-02667-9)
Supplement: Supplementary file 6 — Additional file 6. Risk of bias assessments. [file 13023_2023_2667_MOESM6_ESM.docx]

## **Additional file 6 – Risk of bias assessments**

Joanna Briggs Institute – Text & Opinion (JBI-TO)

| **Study** | **1** | **2** | **3** | **4** | **5** | **6** |
| --- | --- | --- | --- | --- | --- | --- |
| **Behr et al** (2686096) | LR | LR | LR | LR | LR | LR |
| **Banerji et al**  (27661998) | LR | LR | LR | LR | LR | LR |
| **Glassberg**  (29222286) | LR | LR | LR | LR | LR | LR |

1. Is the source of the opinion clearly identified? 2. Does the source of opinion have standing in the field of expertise? 3. Are the interests of the relevant population the central focus of the opinion? 4. Is the stated position the result of an analytical process, and is there logic in the opinion expressed? 5. Is there reference to the extant literature? 6. Is any incongruence with the literature/sources logically defended?

*HR – High risk, MR – Moderate risk, LR – Low risk*

Joanna Briggs Institute – Checklist for prevalence studies (JBI – PS)

| **Study** | **1** | **2** | **3** | **4** | **5** | **6** | **7** | **8** | **9** |
| --- | --- | --- | --- | --- | --- | --- | --- | --- | --- |
| **Pavan et al** (617492938) | I | I | I | I | I | I | I | I | NA |

1. Was the sample frame appropriate to address the target population? 2. Were the study participants sampled in an appropriate way? 3. Was the sample size adequate? 4. Were the study subjects and the setting described in detail? 5. Was the data analysis conducted with sufficient coverage of the identified sample? 6. Were valid methods used for the identification of the condition? 7. Was the condition measured in a standard, reliable way for all participants? 8. Was there appropriate statistical analysis? 9. Was the response rate adequate, and if not, was the low response rate managed appropriately?

*I – Include, E – Exclude, SFI – Seek further information*

Quality Improvement – Minimum Quality Criteria Set (QI-MQCS)

| **Study** | **1** | **2** | **3** | **4** | **5** | **6** | **7** | **8** | **9** | **10** | **11** | **12** | **13** | **14** | **15** | **16** |
| --- | --- | --- | --- | --- | --- | --- | --- | --- | --- | --- | --- | --- | --- | --- | --- | --- |
| **McPhail et al** (20921354) | M | M | M | NM | M | NM | NM | M | M | NM | M | M | M | M | M | M |
| **Moore et al** (22997186) | M | M | M | NM | M | NM | NM | M | M | NM | M | M | M | M | M | M |

1. Organisational motivation 2. Intervention rationale 3. Intervention description 4. Organisational characteristics 5. Implementation 6. Study design 7. Comparator 8. Data source 9. Timing 10. Adherence/Fidelity 11. Health outcomes 12. Organisational readiness 13. Penetration/Reach 14. Sustainability 15. Spread 16. Limitations *M – Met, NM – Not met*

Critical Appraisal Skills Programme (CASP) – Qualitative studies

| **Study** | **1** | **2** | **3** | **4** | **5** | **6** | **7** | **8** | **9** | **10** |
| --- | --- | --- | --- | --- | --- | --- | --- | --- | --- | --- |
| **Maher et al** (28915874) | Y | Y | Y | Y | Y | N | Y | Y | Y | Y |
| **Visser et al** (31691027) | Y | Y | Y | Y | Y | N | Y | Y | Y | Y |

1. Was there a clear statement of the aims of the research? 2. Is a qualitative methodology appropriate? 3. Was the research design appropriate to address the aims of the research? 4. Was the recruitment strategy appropriate to the aims of the research? 5. Was the data collected in the way that addressed the research issue? 6. Has the relationship between researcher and participants been adequately considered? 7. Have ethical issues been taken into account? 8. Was the data analysis sufficiently rigorous? 9. Is there a clear statement of findings? 10. How valuable is the research?

*Y – Yes, CT – Can’t tell, N – No, NA – Not applicable*

Mixed Methods Appraisal Tool (MMAT)

| **Study** | **1** | **2** | **3** | **4** | **5** | **6** |
| --- | --- | --- | --- | --- | --- | --- |
| **Conway et al** (30254788) | LR | LR | MR | MR | MR | MR |
| **Denger et al** (31429780) | LR | LR | LR | LR | LR | LR |
| **Heutinck et al** (33814457) | LR | LR | LR | LR | LR | LR |
| **Johnson et al** (24817340) | LR | LR | LR | LR | LR | LR |
| **Masese et al** (31063506) | LR | LR | LR | LR | LR | LR |
| **Nguyen et al** (20678806) | LR | LR | LR | LR | LR | LR |
| **Utrankar et al** (29741695) | LR | LR | LR | LR | LR | LR |

1. Are there clear research questions? 2. Do the collected data allow to address the research questions? 3. Is the qualitative approach appropriate to answer the research question? 4. Are the qualitative data collection methods adequate to address the research question? 5. Are the findings adequately derived from the data? 6. Is the interpretation of results sufficiently substantiated by data?

*HR – High risk, MR – Moderate risk, LR – Low risk*

Risk of Bias instrument for cross-sectional surveys of attitudes and practices (ROBICSSAP)

| **Study** | **1** | **2** | **3** | **4** | **5** |
| --- | --- | --- | --- | --- | --- |
| **Akiyama et al**  (3259830) | DY | DY | DY | DY | DY |
| **Bashiri et al**  (30842395) | DY | DY | PY | DY | DY |
| **Cabana et al**  (31106244) | DY | PY | PY | DY | DN |
| **Cheung et al**  (27757929) | DY | DN | PY | DY | DN |
| **Fearon et al**  (30387290) | DY | PY | DY | DY | DN |
| **Fu et al**  (29958877) | DY | PY | DY | DY | DN |
| **Garber et al**  (186712474) | DY | PY | PY | DY | DN |
| **Glassberg et al**  (23561465) | DY | DY | DY | DY | PY |
| **Glauser et al**  (22495970) | DY | PY | DY | DY | DY |
| **Harberle & Huemer** (25690729) | DY | DN | DY | DY | DN |
| **Hernandez Trujillo et al** (22670779) | DY | DY | DY | DY | DN |
| **Hernandez Trujillo et al**  (25780256) | DY | PY | DY | DY | DN |
| **Landfeldt et al**  (26870664) | DY | DN | PY | DY | DY |
| **Lanzkron et al**  (18717150) | DY | DN | PY | DY | DN |
| **Lunyera et al**  (27506442) | DY | DN | PY | DY | DN |
| **Mackenzie et al**  (9861225) | DY | PY | DY | DY | DN |
| **Nanda et al**  (24928306) | DY | DY | PY | DY | DN |
| **Orange et al**  (27066486) | DY | PN | DY | DY | DN |
| **Piekert et al**  (18621518) | DY | DN | DY | DY | DY |
| **Riedl et al**  (21457880) | DY | PN | DY | DY | DN |
| **Riedl et al**  (33122123) | DY | PN | DY | DY | DN |
| **Riedl et al**  (25609328) | DY | PN | DY | DY | DN |
| **Robalo-Cordeiro & Morais**  (32561352) | DY | PY | DY | DY | DN |
| **Spickett et al**  (10156396) | DY | DY | DY | DY | DN |
| **Talarico et al**  (32868449) | DY | PY | PY | DY | DN |
| **Vry et al**  (27911335) | DY | PY | PY | DY | DN |
| **Wurst & Sleath**  (15150156) | DY | PY | PY | DY | PY |
| **Yong et al**  (19914873) | DY | PY | DY | DY | DN |

1. Is the source population representative of the population of interest? 2. Is the response rate adequate? 3. *Absence* of missing data? 4. Is the survey clinically sensible? 5. Is there any evidence for the reliability and validity of the survey instrument?

*DY – Definitely yes, PY – Probably yes, PN – Probably no, DN – Definitely No*

Risk of Bias in Systematic Reviews (ROBIS)

| **Study** | 1 | 2 | 3 | 4 | 5 | 6 | 7 | 8 | 9 | 10 | 11 | 12 | 13 | 14 | 15 | 16 | 17 | 18 | 19 | 20 | 21 |
| --- | --- | --- | --- | --- | --- | --- | --- | --- | --- | --- | --- | --- | --- | --- | --- | --- | --- | --- | --- | --- | --- |
| **Corrigan et al** (31321066) | Y | Y | Y | Y | Y | Y | Y | Y | Y | PY | PY | Y | Y | N | N | Y | Y | Y | PN | NI | N |

1. Did the review adhere to pre-defined objectives and eligibility criteria? 2. Were the eligibility criteria appropriate for the review question? 3. Were eligibility criteria unambiguous? 4. Were any restrictions in eligibility criteria based on study characteristics appropriate? 5. Were any restrictions in eligibility criteria based on sources of information appropriate? 6. Did the search include an appropriate range of databases/electronic sources for published and unpublished reports? 7. Were methods additional to database searching used to identify relevant reports? 8. Were the terms and structure of the search strategy likely to retrieve as many eligible studies as possible? 9. Were restrictions based on date, publication format, or language appropriate? 10. Were efforts made to minimise error in selection of studies? 11. Were efforts made to minimise error in data collection? 12. Were sufficient study characteristics available for both review authors and readers to be able to interpret the results? 13. Were all relevant study results collected for use in the synthesis? 14. Was risk of bias (or methodological quality) formally assessed using appropriate criteria? 15. Were efforts made to minimise error in risk of bias assessment? 16. Did the synthesis include all studies that it should? 17. Were all pre-defined analyses reported or departures explained? 18. Was the synthesis appropriate given the nature and similarity in the research questions, study designs and outcomes across included studies? 19. Was between-study variation (heterogeneity) minimal or addressed in the synthesis? 20. Were the findings robust e.g. as demonstrated through funnel plot or sensitivity analyses? 21. Were biases in primary studies minimal or addressed in the synthesis?

*Y – Definitely yes, PY – Probably yes, PN – Probably no, N – No, NI – No information*
