# Supplementary material for: Barriers and facilitators to the implementation of guidelines in rare diseases: a systematic review
Source: Orphanet J Rare Dis. 2023 Jun 7;18:140. doi: 10.1186/s13023-023-02667-9 (PMC10246545; doi:10.1186/s13023-023-02667-9)
Supplement: Supplementary file 7 — Additional file. 7 Determinants of Practice. [file 13023_2023_2667_MOESM7_ESM.docx]

## **Additional file 7 – Determinants of Practice**

|  |  | **Number of studies** | | |
| --- | --- | --- | --- | --- |
|  |  | **Barrier** | **Facilitator** | **Both** |
| **Flottorp Domains** | | **168** | **52** | **29** |
| **Domain 1 - Guideline factors** | | **39** | **8** | **1** |
| 1.1 | Recommendation | 22 | 6 | 1 |
| 1.1.1 | Quality of evidence supporting the recommendation | 6 | 0 | 0 |
| 1.1.2 | Strength of recommendation | 1 | 0 | 0 |
| 1.1.3 | Clarity | 5 | 1 | 0 |
| 1.1.4 | Cultural appropriateness | 1 | 0 | 1 |
| 1.1.5 | Accessibility of the recommendation | 6 | 4 | 0 |
| 1.1.6 | Source of the recommendation | 2 | 1 | 0 |
| 1.1.7 | Consistency with other guidelines | 1 | 0 | 0 |
| 1.2 | Recommended clinical intervention | 16 | 1 | 0 |
| 1.2.1 | Feasibility | 11 | 1 | 0 |
| 1.2.2 | Accessibility of the intervention | 5 | 0 | 0 |
| 1.3 | Recommended behaviour | 1 | 1 | 0 |
| 1.3.1 | Compatibility | 1 | 0 | 0 |
| 1.3.2 | Effort | 0 | 0 | 0 |
| 1.3.3 | Trialability | 0 | 0 | 0 |
| 1.3.4 | Observability | 0 | 1 | 0 |
| **Domain 2 - Individual health professional factors** | | **61** | **20** | **17** |
| 2.1 | Knowledge and skills | 35 | 11 | 14 |
| 2.1.1 | Domain knowledge | 15 | 2 | 7 |
| 2.1.2 | Awareness and familiarity with the recommendation | 15 | 8 | 5 |
| 2.1.3 | Knowledge about own practice | 0 | 1 | 1 |
| 2.1.4 | Skills needed to adhere | 5 | 0 | 1 |
| 2.2 | Cognitions (including attitudes) | 25 | 9 | 3 |
| 2.2.1 | Agreement with the recommendation | 7 | 5 | 3 |
| 2.2.2 | Attitudes towards guidelines in general | 2 | 1 | 0 |
| 2.2.3 | Expected outcome | 7 | 1 | 0 |
| 2.2.4 | Intention and motivation | 3 | 1 | 0 |
| 2.2.5 | Self-efficacy | 4 | 1 | 0 |
| 2.2.6 | Learning style | 0 | 0 | 0 |
| 2.2.7 | Emotions | 2 | 0 | 0 |
| 2.3 | Professional behaviour | 1 | 0 | 0 |
| 2.3.1 | Nature of the behaviour | 1 | 0 | 0 |
| 2.3.2 | Capacity to plan change | 0 | 0 | 0 |
| 2.3.3 | Self-monitoring or feedback | 0 | 0 | 0 |
| **Domain 3 - Patient factors** | | **20** | **10** | **7** |
| 3.1 | Patient needs | 4 | 3 | 1 |
| 3.2 | Patient beliefs and knowledge | 4 | 2 | 2 |
| 3.3 | Patient preferences | 5 | 1 | 2 |
| 3.4 | Patient motivation | 2 | 2 | 1 |
| 3.5 | Patient behaviour | 5 | 2 | 1 |
| **Domain 4 - Professional interactions** | | **12** | **5** | **2** |
| 4.1 | Communication and influence | 6 | 2 | 1 |
| 4.2 | Team processes | 3 | 0 | 1 |
| 4.3 | Referral processes | 3 | 3 | 0 |
| **Domain 5 - Incentives and resources** | | **16** | **5** | **2** |
| 5.1 | Availability of necessary resources | 9 | 0 | 0 |
| 5.2 | Financial incentives and disincentives | 1 | 1 | 1 |
| 5.3 | Nonfinancial incentives and disincentives | 0 | 0 | 1 |
| 5.4 | Information system | 1 | 3 | 0 |
| 5.5 | Quality assurance and patient safety systems | 0 | 0 | 0 |
| 5.6 | Continuing education system | 0 | 0 | 0 |
| 5.7 | Assistance for clinicians | 5 | 1 | 0 |
| **Domain 6 - Capacity for organisational change** | | **0** | **0** | **0** |
| 6.1 | Mandate, authority and accountability | 0 | 0 | 0 |
| 6.2 | Capable leadership | 0 | 0 | 0 |
| 6.3 | Relative strength of supporters and opponents | 0 | 0 | 0 |
| 6.4 | Regulations, rules and policies | 0 | 0 | 0 |
| 6.5 | Priority of necessary change | 0 | 0 | 0 |
| 6.6 | Monitoring and feedback | 0 | 0 | 0 |
| 6.7 | Assistance for organisational changes | 0 | 0 | 0 |
| **Domain 7 - Social, political and legal factors** | | **20** | **4** | **0** |
| 7.1 | Economic constraints on the healthcare budget | 8 | 1 | 0 |
| 7.2 | Contracts | 0 | 0 | 0 |
| 7.3 | Legislation | 2 | 0 | 0 |
| 7.4 | Payer or funder policies | 9 | 1 | 0 |
| 7.5 | Malpractice liability | 1 | 0 | 0 |
| 7.6 | Influential people | 0 | 2 | 0 |
| 7.7 | Corruption | 0 | 0 | 0 |
| 7.8 | Political stability | 0 | 0 | 0 |
