# Supplementary material for: Barriers and facilitators to the implementation of guidelines in rare diseases: a systematic review
Source: Orphanet J Rare Dis. 2023 Jun 7;18:140. doi: 10.1186/s13023-023-02667-9 (PMC10246545; doi:10.1186/s13023-023-02667-9)
Supplement: Supplementary file 9 — Additional file 9. Overview of the contribution of individual studies. [file 13023_2023_2667_MOESM9_ESM.docx]

## **Additional file 9 – Overview of the contribution of individual studies**

**KEY:** B - Barrier F - Facilitator N – Neutral

**KEY:** B - Barrier F - Facilitator N - Neutral
